# Meta-Analysis Derived (MAD) Transcriptome of Psoriasis Defines the “Core” Pathogenesis of Disease

**DOI:** 10.1371/journal.pone.0044274

**Published:** 2012-09-05

**Authors:** Suyan Tian, James G. Krueger, Katherine Li, Ali Jabbari, Carrie Brodmerkel, Michelle A. Lowes, Mayte Suárez-Fariñas

**Affiliations:** 1 Center for Clinical and Translational Science, The Rockefeller University, New York, New York, United States of America; 2 Laboratory for Investigative Dermatology, The Rockefeller University, New York, New York, United States of America; 3 Immunology & Biomarkers, Janssen Research & Development, Radnor, Pennsylvania, United States of America; The University of Queensland, Australia

## Abstract

The cause of psoriasis, a common chronic inflammatory skin disease, is not fully understood. Microarray experiments have been widely used in recent years to identify genes associated with psoriasis pathology, by comparing expression levels of lesional (LS) with adjacent non-lesional (NL) skin. It is commonly observed that the differentially expressed genes (DEGs) differ greatly across experiments, due to variations introduced in the microarray experiment pipeline. Therefore, a statistically based meta-analytic approach, which combines the results of individual studies, is warranted. In this study, a meta-analysis was conducted on 5 microarray data sets, including 193 LS and NL pairs. We termed this the Meta-Analysis Derived (MAD) transcriptome. In “MAD-5” transcriptome, 677 genes were up-regulated and 443 were down-regulated in LS skin compared to NL skin. This represents a much larger set than the intersection of DEGs of these 5 studies, which consisted of 100 DEGs. We also analyzed 3 of the studies conducted on the Affymetrix hgu133plus2 chips and found a greater number of DEGs (1084 up- and 748 down-regulated). Top canonical pathways over-represented in the MAD transcriptome include *Atherosclerosis Signaling* and *Fatty Acid Metabolism*, while several “new” genes identified are involved in Cardiovascular Development and Lipid Metabolism. These findings highlight the relationship between psoriasis and systemic manifestations such as the metabolic syndrome and cardiovascular disease. Then, the Meta Threshold Gradient Descent Regularization (MTGDR) algorithm was used to select potential markers distinguishing LS and NL skin. The resulting set (20 genes) contained many genes that were part of the residual disease genomic profile (RDGP) or “molecular scar” after successful treatment, and also genes subject to differential methylation in LS tissues. To conclude, this MAD transcriptome yielded a reference list of reliable psoriasis DEGs, and represents a robust pool of candidates for further discovery of pathogenesis and treatment evaluation.

## Introduction

Psoriasis vulgaris is a common chronic inflammatory skin disease of varying severity, characterized by red scaly plaques. The pathogenesis of psoriasis has well recognized contributions from the skin, immune system, and genetic factors. With increased validation of microarray technology, microarrays have become a valuable tool to explore the pathogenesis of psoriasis and to elucidate the mechanisms of action of promising treatments. Using microarray experiments, several groups have defined lists of differentially expressed genes (DEG) between lesional (LS) versus uninvolved or non-lesional (NL) skin of psoriasis patients [1]–[8]. Such lists of DEGs may serve as foundation for the purpose of defining the psoriasis transcriptome and explaining pathology [2], [4], as well as characterizing treatment responses [9], and residual disease after treatment [10], [11].

The most common approach to synthesize published transcriptomes is to intersect and visualize them through Venn-diagrams. However it is frequently observed that DEG lists produced by different experiments differ for a plethora of conditions including variations in the phenotype of the disease itself [6]. This leads to a very narrow intersection and raises doubts about the existence of a disease core. A comprehensive review on the existence of this large discordance was given by Cahan *et al.* [12], and the authors summarized three major sources accounting for this discordance: variation from random noise, biological and experimental differences, and differences in technical methods. Suarez-Farinas *et al.* [6] used Gene Set Enrichment Analysis (GSEA) to validate a new list of DEGs of a microarray study, rather than the Venn-diagram approach. GSEA provides a quick tool to assess if a new experiment is in agreement with previously published studies. However, it does not address the goal of obtaining the common molecular features of psoriasis across different labs, patient populations, and with a variety of disease severity.

To combine results of individual studies and obtain a list of more “robust” DEGs with a reliable estimation of the effect size considering the above-mentioned variations, a statistically based meta-analytic approach is recommended [13]. Formally, meta-analysis refers to an integrative data analysis method that is defined as a synthesis of results from datasets that are independent but related [14]. Such a method has ranging benefits as summarized by Campaign and Yang [15]. Meta-analysis produces overall effect estimates with considerably more statistical power than individual studies. Statistical power improves with an increase in sample size of the combined studies, and hence, there is an increase in the ability to find true effects that are missed by any individual study. Moreover, meta-analysis alleviates conflicting results obtained by separate studies as it estimates overall average effects and focuses on the variations between phenotypes. Hence, meaningful effects and relationships upon which studies agree are more likely to be discovered by meta-analysis than by less systematic and analytic approaches.

Here, a meta-analysis was conducted using microarray data from 5 studies [2], [4], [6]–[8] consisting of 386 paired-samples from 193 patients. The raw data (CEL files) were obtained from a public repository, and the same preprocessing and analytic procedures were followed across all studies. A meta-analytic model was used to compare gene expression profiles of LS samples with their paired NL biopsies across studies, and an overall estimation of the fold changes (FCH) was estimated and the statistical significance was assessed. Using this approach, we produced a list of DEGs that represent a robust reference psoriasis transcriptome, which we have termed **M**eta-**A**nalysis **D**erived, or MAD, transcriptome.

## Results

**Figure 1:**
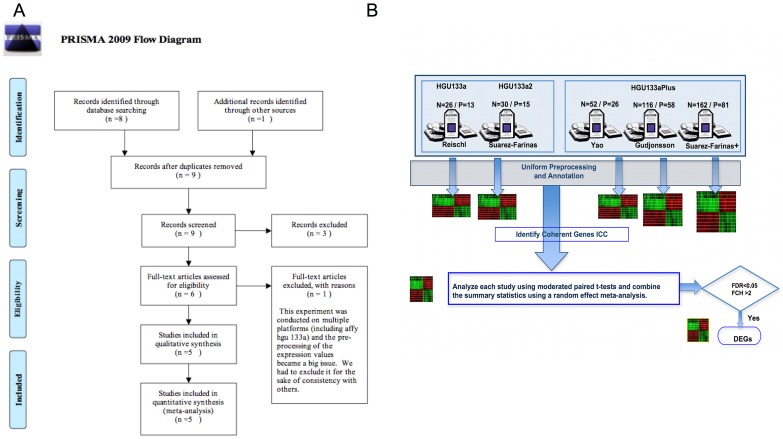
PRIMA diagram and study schema. A. PRIMA (Preferred Reporting Items for Systematic Reviews and Meta-Analyses) diagram. B. Schema describing the steps taken during the meta-analysis. N and P represent the number of samples (N) and patients (P) respectively in each study.

Figure 1 shows the overall study flow for this meta-analysis, and a summary of the 5 studies is given in Table S1. Following the steps given in Figure 1, the results from the meta-analysis are described below.

### Coherence among Studies and Selection of Coherent Genes

First, a general agreement of microarray raw data produced by different studies was estimated using Integrated Correlation Analysis [16]. This method produces a general coefficient called Integrated Correlation Coefficient (ICC), with similar interpretations as the Pearson correlation coefficient, which represents agreement between studies. Additionally, ICC can be used to eliminate background noise prior to the analysis, excluding genes that exhibit incoherent behavior across studies (see Materials and Methods for details).

For 14483 probe-sets (9264 genes) that passed the first-step filtering (see Materials and Methods for details) and were then common among the 5 studies, the ICC was calculated as 0.406 (95% CI: [0.402, 0.409]). When genes with poor coherent behavior (as defined by those on the lower first quantile of the distribution) were filtered out, an improvement on the ICC to 0.569 (95% CI: [0.565, 0.573]) was observed. The resulting 10862 probe sets (7534 genes) were used for downstream analysis.

### Model Specification

A random effect meta-analysis model was used to analyze the expression differences between LS and NL samples. The choice of using a random or a fixed effect meta-analysis was based on the comparison between sample quantiles of Cochran’s Q and the quantiles of its theoretical distribution (χ^2^_n−1_, where n represents the number of studies) as suggested by Choi *et al.* [17]. The QQ-plot (Figure S1A) shows a substantial deviation in Cochran’s Q from the desired distribution indicating that a random effect model is more appropriate. Comparing the standardized overall effect estimates from the random effect meta-analysis model to a standard normal distribution shows that those estimates do not deviate dramatically from normality (Figure S1B).

### The MAD Transcriptome in Psoriasis

The meta-analysis of 5 studies allowed us to estimate the overall difference in expression values between LS and NL samples across studies (193 LS and NL pairs). Using FDR<0.05 and FCH>2, which were the same cut-offs for all the published studies, we identified 854 up-regulated and 550 down-regulated probe-sets (Table S2) representing 677 and 443 known unique genes, respectively (by ENTREZ identifiers). We refer to this transcriptome as MAD-5.

A microarray meta-analysis is restricted to the universe of genes commonly present on each chip platform used for sample hybridization. The hgu133plus2 chips contain more than twice the number of probe-sets than the hgu133a2 chips, representing 7315 genes whose effect size cannot therefore be assessed by MAD-5, and which may be biologically relevant. Therefore the same analysis was carried out considering the 163 LS and NL pairs from the 3 studies that used hgu133plus2 chips. Using 25% cutoff for coherence scores, 24375 probe sets (9222 genes) were considered for downstream analysis. The transcriptome for 133plus2 (MAD-3) encompassed 1412 up-regulated and 959 down-regulated probe-sets (Table S3) representing 1084 and 748 genes, respectively, a list considerably larger than the MAD-5 transcriptome.

**Figure 2:**
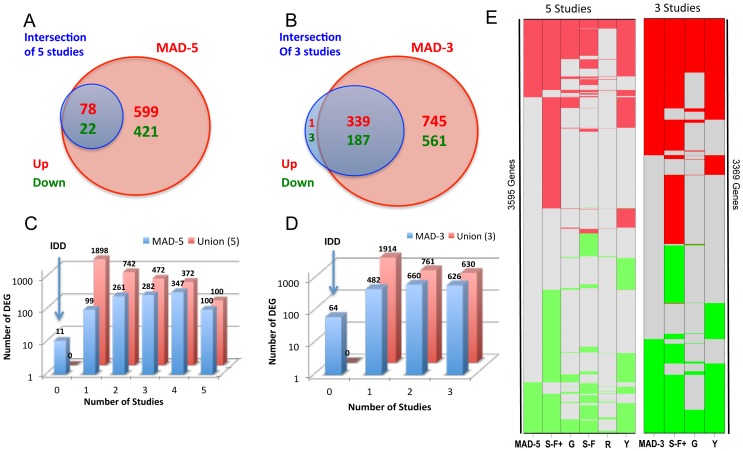
Overview of the MAD-5 and MAD-3 transcriptomes. A. Venn diagram showing that when comparing the MAD-5 transcriptome with the intersection of DEGs identified by individual studies, the meta-analysis always identified a much larger set. B. Venn diagram showing the same comparison as A but for MAD-3 transcriptome. C. 3D Barplots showing the overlap of MAD-5 genes (blue bars) by the number of individual studies (x-axis). For example: among the MAD-5 transcriptome, 347 genes were identified by 4 studies and 100 by all 5 studies. For comparison the numbers for the set of genes that were identified by any of the individual studies (Union) is also represented (red bars). Most DEGs from the meta-analysis appeared in at least two of these studies. Integration Discovery Genes (IDD) represents the set of genes only identified by the meta-analysis. D. 3D Barplots showing the same comparison as C for MAD-3. E. Color-coded graphs showing the comparison of MADs transcriptomes and individual studies. Each row represents a gene and the color indicates whether the gene is up-regulated (red), down-regulated (green) or not differentially expressed (gray) in each (columns) and the meta-analysis. Meta: meta-analysis; S-F+: Suarez-Farinas 2012, hgu33plus2 chips; G: Gudjonsson’2009; S-F: Suarez-Farinas’2010; R: Reischl’2007; Y: Yao’2008.

The intersection of DEGs reported by the 5 individual studies consisted of 78 up- and 22 down-regulated genes. However, the global psoriasis transcriptome obtained by the MAD-5 is much larger than this intersection (Figure 2A) and successfully identified those 100 genes. When only hgu133plus2-studies were considered, 340 up- and 190 down-regulated genes were in the intersection, and all but 4 (0.75%) genes were identified by the meta-analysis (Figure 2B). A simplified heat-map is presented in Figure 2E, showing how the DEGs in each individual study relate with those identified by the meta-analyses.

The 4 genes from the intersection that were not identified by the MAD-3 were IDA, LEPR, MYOCD and TYMP. In the meta-analysis several factors intervene in the estimation of the overall summary statistics for each gene: the log-fold change (LFC), its corresponding standard deviation in each study, and also the between-study variance (denoted by τ^2^ in Equation 1). Large values of τ^2^ will lead to a smaller overall LFC and overall T statistics such as in the case of the four above-mentioned genes. Those 4 genes have p<0.056 and FDR<0.085 values, and a less strict FDR cut-off would include them.

It can be observed that the number of DEGs in the meta-analyses is in between the numbers of DEGs from separate studies (Figure 2E). Not surprisingly, the number of significant genes identified by the meta-analysis was smaller than some of the individual studies, namely Yao’s and Suarez-Farinas+ (Figure 2E). The meta-analytic approach depicted here is concerned with genes that were commonly dysregulated among all the studies and with consistent behavior (evaluated by coherence score), so fewer genes were considered in the meta-analysis compared with individual studies. Additionally, outliers in individual studies with smaller sample size can easily influence the gene detection. Since the overall estimates in a meta-analysis are essentially weighted averages of each individual study with the weights setting as the precision of each study, the influence of a single study is attenuated in the meta-analysis approach. Thus it is expected that most DEGs in the meta-analysis appear in at least two out of the five studies. Figures 2C and 2D illustrate this point, and show that the resultant list is a more concordant representative of the DEGs in a larger patient population encompassing different laboratory settings.

Although the analysis of MAD-5, which involves samples from 5 labs, is statistically more robust, we will feature the MAD-3 transcriptome, which has a greater number of potential genes. Results and insights derived from MAD-3 are presented in this manuscript, while results from MAD-5 are provided as Table S2. Table 1 represents the top 25 up-and down-regulated genes in MAD-3 (Table S3), with fold-changes estimated by the meta-analysis, ranging from 33 to more than 600. As expected, the top 25 up- and down-DEGs were all identified by the hgu133plus2 previously published transcriptomes. Additionally, 21 up- and 13 down- regulated genes from MAD-3 were also among the top 25 up- and down-regulated genes in the MAD-5 transcriptome. Overall, the MAD-3 transcriptome contains well-recognized psoriasis genes in the “top” up- and down-regulated gene lists, and represents a meaningful list of DEGs to further evaluate, as discussed below.

**Table 1:** Top 25 Up and Down-regulated genes in the MAD-3 transcriptome.

	Probe	Symbol	Description	RefSeq	LFC	FC
**Up-regulated genes**						
1	211906_s_at	SERPINB4	serpin peptidase inhibitor, clade B (ovalbumin), member 4	6318	9.37	660.69
2	205863_at	S100A12	S100 calcium binding protein A12	6283	8.36	328.42
3	205513_at	TCN1	transcobalamin I (vitamin B12 binding protein, R binder family)	6947	8.27	309.43
4	232170_at	S100A7A	S100 calcium binding protein A7A	338324	8.02	259.85
5	220664_at	SPRR2C	small proline-rich protein 2C (pseudogene)	6702	7.38	167.06
6	207356_at	DEFB4A	defensin, beta 4A	1673	7.1	137.64
7	206561_s_at	AKR1B10	aldo-keto reductase family 1, member B10 (aldose reductase)	57016	6.48	89.07
8	41469_at	PI3	peptidase inhibitor 3, skin-deri_ved	5266	6.32	79.69
9	202859_x_at	IL8	interleukin 8	3576	6.04	65.85
10	207602_at	TMPRSS11D	transmembrane protease, serine 11D	9407	5.97	62.8
11	209720_s_at	SERPINB3	serpin peptidase inhibitor, clade B (ovalbumin), member 3	6317	5.96	62.22
12	203535_at	S100A9	S100 calcium binding protein A9	6280	5.9	59.78
13	205660_at	OASL	2′-5′-oligoadenylate synthetase-like	8638	5.8	55.89
14	207367_at	ATP12A	ATPase, H+/K+ transporting, nongastric, alpha polypeptide	479	5.75	53.78
15	212531_at	LCN2	lipocalin 2	3934	5.74	53.31
16	219554_at	RHCG	Rh family, C glycoprotein	51458	5.7	51.98
17	207602_at	IGFL1	IGF-like family member 1	374918	5.6	48.36
18	217388_s_at	KYNU	kynureninase (L-kynurenine hydrolase)	8942	5.58	48
19	220322_at	IL1F9	interleukin 1 family, member 9	56300	5.44	43.45
20	204733_at	KLK6	kallikrein-related peptidase 6	5653	5.43	43.05
21	202018_s_at	LTF	lactotransferrin	4057	5.17	36.11
22	205476_at	CCL20	chemokine (C-C motif) ligand 20	6364	5.13	34.92
23	227736_at	C10orf99	chromosome 10 open reading frame 99	387695	5.07	33.6
24	219403_s_at	HPSE	heparanase	10855	5.07	33.48
25	206134_at	ADAMDEC1	ADAM-like, decysin 1	27299	5.05	33.15
**Down-regulated genes**						
1	204712_at	WIF1	WNT inhibitory factor 1	11197	−4.16	−17.88
2	205404_at	HSD11B1	hydroxysteroid (11-beta) dehydrogenase 1	3290	−3.31	−9.92
3	207955_at	CCL27	chemokine (C-C motif) ligand 27	10850	−3.28	−9.71
4	227174_at	WDR72	WD repeat domain 72	256764	−3.23	−9.38
5	205883_at	ZBTB16	zinc finger and BTB domain containing 16	7704	−3.18	−9.06
6	237120_at	KRT77	keratin 77	374454	−3.17	−9.00
7	207326_at	BTC	betacellulin	685	−3.17	−9.00
8	210297_s_at	MSMB	microseminoprotein, beta-	4477	−3.11	−8.63
9	214240_at	GAL	galanin prepropeptide	51083	−3.02	−8.11
10	239929_at	PM20D1	peptidase M20 domain containing 1	148811	−2.94	−7.67
11	214598_at	CLDN8	claudin 8	9073	−2.8	−6.96
12	224555_x_at	IL1F7	interleukin 1 family, member 7 (zeta)	27178	−2.79	−6.92
13	205030_at	FABP7	fatty acid binding protein 7, brain	2173	−2.78	−6.87
14	217059_at	MUC7	mucin 7, secreted	4589	−2.68	−6.41
15	205979_at	SCGB2A1	secretoglobin, family 2A, member 1	4246	−2.67	−6.36
16	234513_at	ELOVL3	elongation of very long chain fatty acids (FEN1/Elo2, SUR4/Elo3, yeast)-like 3	83401	−2.67	−6.36
17	1554195_a_at	C5orf46	chromosome 5 open reading frame 46	389336	−2.64	−1.00
18	235278_at	MACROD2	MACRO domain containing 2	140733	−2.61	−6.23
19	239547_at	HS3ST6	heparan sulfate (glucosamine) 3-O-sulfotransferase 6	64711	−2.58	−6.11
20	204607_at	HMGCS2	3-hydroxy-3-methylglutaryl-CoA synthase 2 (mitochondrial)	3158	−2.51	−5.98
21	239017_at	COL6A4P1	collagen, type VI, alpha 4 pseudogene 1	344875	−2.5	−5.70
22	1553583_a_at	THRSP	thyroid hormone responsive	7069	−2.46	−5.66
23	213661_at	PAMR1	peptidase domain containing associated with muscle regeneration 1	25891	−2.35	−5.50
24	223836_at	FGFBP2	fibroblast growth factor binding protein 2	83888	−2.33	−5.10
25	227803_at	ENPP5	ectonucleotide pyrophosphatase/phosphodiesterase 5 (putative)	59084	−2.25	−5.03

### Primary Cytokines in MAD Psoriasis

In Suarez-Farinas *et al.* [6], we draw attention to the fact that many of the primary cytokines known to be elevated in psoriasis which are considered excellent therapeutic targets, were not detected by microarray approaches (Table 3, [6]). This is primarily due to a limitation of the platform since fold-changes are underestimated for low-abundance transcripts. This observation holds true in both MAD-3 and MAD-5, which did not identify p19, p40, LTA1, IL-22, IFNγ, IL-4, IL-6, iNOS, p35, and CCL3. IL-17, IL20 and CCL4 were identified by the MAD-3 but their overall fold changes (2.8, 2.36 and 2.06) were much smaller than the RT-PCR based FCH (6.2, 4 and 2.8 respectively) reported in Table 3 of [6]. To overcome this limitation, Nograles *et al.* defined the genomic response to IL-17, TNF, IL-22 and INFγ in keratinocytes [18] and we have used them in many mechanistic studies. Using GSEA approaches, those cytokines pathways were up-regulated in psoriasis [6], [8]. In the MAD-3, Normalized Enrichment Scores (NES) for these cytokine-induced keratinocyte “pathways” or gene sets were: 2.19 for IL-17 genes, 2.04 for TNF, 2.11 for IL-22 and 2.41 for IFNγ (FDR<0.0001 in all cases). Genes with a synergistic response to IL-17 and TNF [19] were also enriched (NES = 2.83, FDR<0.001) in the MAD-3 transcriptome. Hence, as anticipated, the hallmark cytokines products were represented in the meta-analysis, even though the primary cytokines were difficult to detect.

### Cutaneous Compartment Localization of the MAD Transcriptome

**Figure 3:**
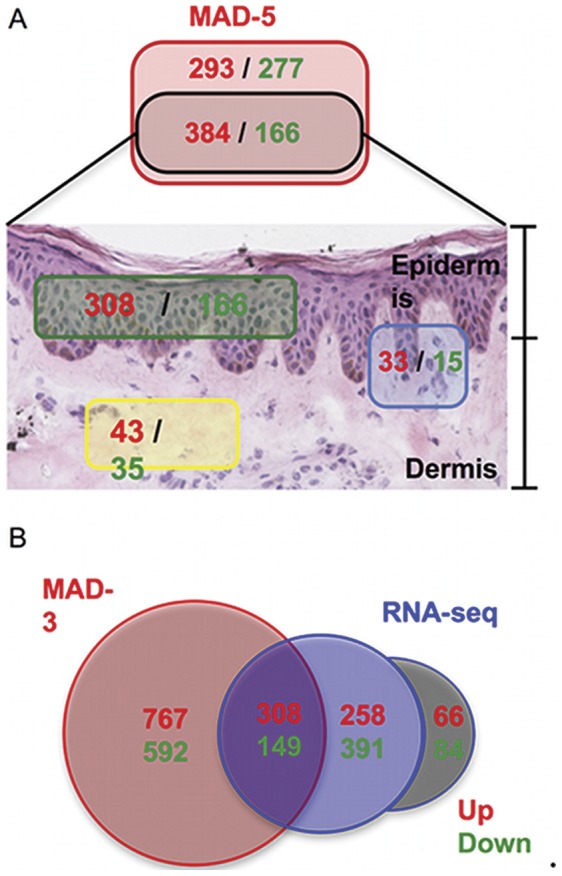
Comparison with other transcriptomes. A. Cutaneous localization of MAD-5 transcriptome. 49% of MAD-5 genes (black rectangle) were identified as being part of the Epidermis (green rectangle) or Dermis (yellow rectangle) psoriasis transcriptome defined by the use of laser capture micro-dissection (LCM) techniques (using the same cutoffs FDR<0.05 and FCH>2). Genes identified in both Epidermis and Dermis transcriptomes are in the blue rectangle. B. Venn-Diagram showing the intersection between the MAD-3 psoriasis transcriptome and RNA-seq pilot experiment (using the same cutoffs FDR<0.05 and FCH>2). Numbers are colored in red and green to represent up-regulated or down-regulated genes respectively. The gray zone represents genes identified by RNA-seq but that were not physically present in the hgu133plus2 chips.

Mitsui *et al*. [20] performed a laser capture micro-dissection (LCM) study with psoriatic NL and LS skin (n = 3), comparing gene expression profiles between NL and LS. Using this method they generated a transcriptome for the epidermis as well as the dermis with the same cutoffs for FCH and FDR as in the meta-analysis. The use of LCM may increase the sensitivity of detecting DEGs even in the presence of a small sample size. We compared the MAD-5 transcriptome in this case, as the LCM-generated transcriptomes were on the hgu133a2 chips. 49% of the up regulated MAD-5 DEGs were identified in either the epidermis or dermis by LCM, as shown in Figure 3A, even though the LCM study was underpowered. Nevertheless, this offered useful information into the cutaneous localization of the MAD transcriptome, which has been included in Tables S2 and S3.

### Comparisons with Pilot RNA-Seq Study

The MAD-3 transcriptome presented here (with the maximal number of available probes on the hgu133aplus2 chip) was also compared with a pilot RNA-seq study conducted by Jabbari *et al.* [21] using LS and NL samples from 3 patients (Figure 3B). The major advantages of mRNA sequencing-based expression profiling are its deep coverage and large dynamic range of expression levels over which transcripts can be detected. Using the same cutoffs for FCH and FDR as in the meta-analysis, the RNA-seq study identified 1343 DEGs. RNA-sequencing can potentially detect any gene in a sample, so 37% (498/1343, gray area of Figure 3B) of genes identified by RNA-seq could not possibly be identified by meta-analysis because they were not physically present on the hgu133plus2 chips. Of those 845 RNA-seq genes represented on the chip, the MAD-3 transcriptome identified 467 (55.3%). Many more genes were identified by the meta-analysis (1365) than the RNA-seq analysis, which can be attributed to the lack of power of the RNA-seq study, emphasizing the need for larger sample size on RNA-seq studies to make this technology worthwhile.

### Ingenuity Pathway Analysis (IPA)

IPA was used to identify pathways, functions and diseases significantly overrepresented in the MAD-3 transcriptome. Significant pathways and networks enriched in the MAD-3 transcriptome with a FDR<0.05 are presented in Table S4. Given recent emphasis on the relationship between psoriasis and systemic manifestations such as the metabolic syndrome [22], it is pertinent that the top canonical pathway was *Atherosclerosis Signaling*, and *Fatty Acid Metabolism* was also among the top ten significant pathways. *Cancer, Cardiac hyperplasia/hyperproliferation and Cardiovascular Disease* were also top networks. IL-17A was a key cytokine represented in the overlapping networks, although this primary cytokine was barely detected directly by MAD-3. IL-17-related pathways were highly represented, with five canonical pathways in the top 40 containing IL-17. Top canonical pathways representing the link between the innate and adaptive immunity, were also present, such as *Dendritic Cell Maturation, Role of Cytokines in Mediating Communication between Immune Cells, Communication between Innate and Adaptive Immune Cells,* and *Fcγ Receptor-mediated Phagocytosis in Macrophages and Monocytes.*

*Interferon Signaling* and *Role of JAK1 and JAK3 in γc Cytokine Signaling* were both represented in the top canonical pathways. Many other cytokine pathways were also significant, paralleling the cytokine-rich environment in psoriasis, including *IL-10, IL-12, IL-2, IL-9, IL-22, IL-15, IL-6,* and *IL-8 Signaling*. The *Production of Nitric Oxide and Reactive Oxygen Species in Macrophages* pathway was also significant, which is relevant since there is an abundance of TNF- and iNOS-producing dendritic cells (TIP-DCs), also called inflammatory myeloid DCs, present in psoriasis lesions [23]. The identification of *IL-12 Signaling and Production in Macrophages* pathway is also interesting given the presence of genetic single nucleotide polymorphisms (SNPs) in the IL-12/IL-23 system in psoriasis [24], [25].

**Figure 4:**
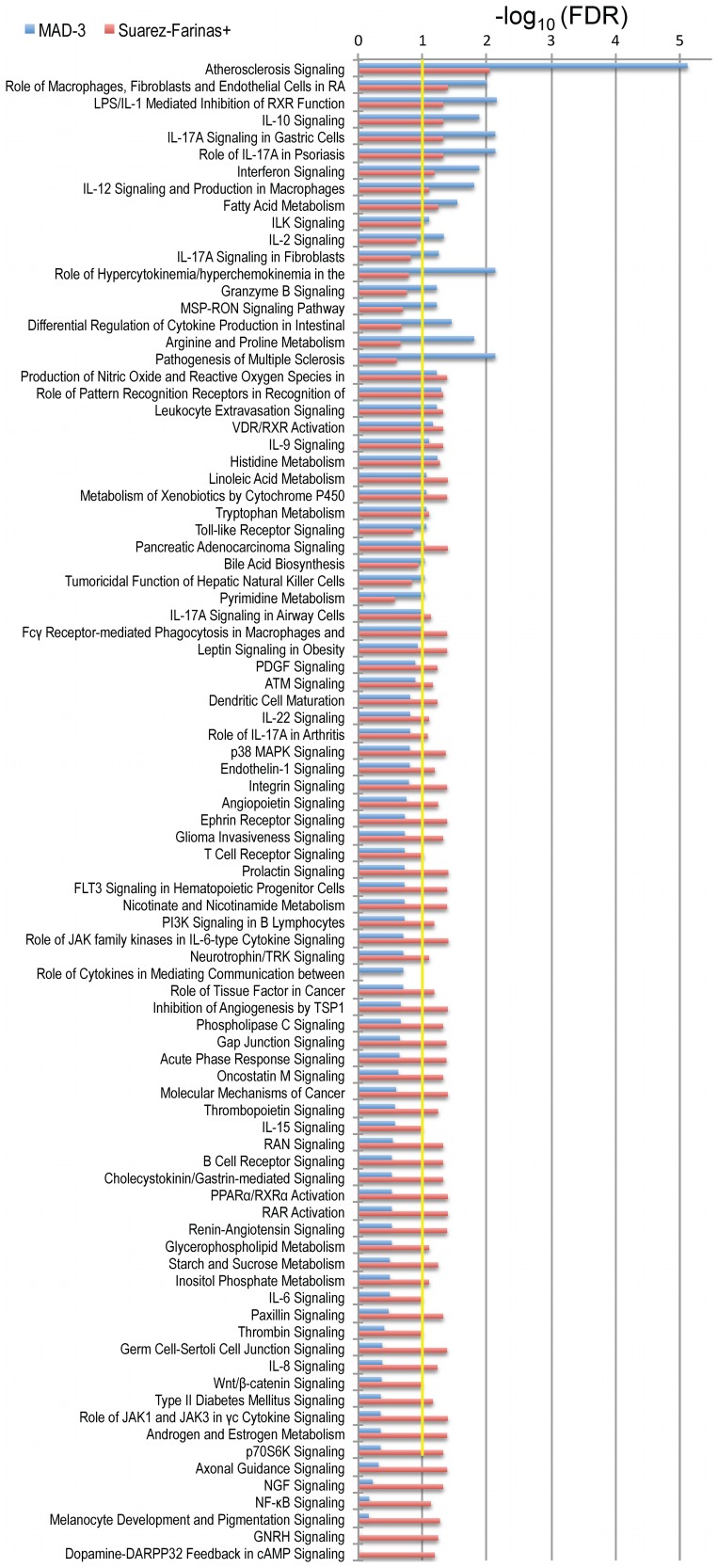
Ingenuity Pathway Analysis. Comparison of canonical pathways overrepresented in MAD-3 transcriptome (blue bars) and Suarez-Farinas^+^ (red bars), which is the study with the largest sample size and number of DEG. Bars represents a –log_10_ transformation of the Benjamini-Hochberg adjusted p-value, which controls FDR. Only pathways with FDR<0.1 (which corresponds to 1 in the –log_10_ scale; represented by yellow line) in either MAD-3 or Suarez-Farinas^+^ are shown.

The strength of the association of the canonical pathways in MAD-3 transcriptome was compared with that of the Suarez-Farinas+ transcriptome (Figure 4), which is the largest data-set published to date with the greatest number of psoriasis DEGs [8]. As is shown in Figure 4, all the commonly recognized pathways in psoriasis were over-represented in both the MAD-3 and Suarez-Farinas+ transcriptomes, but the association was stronger in the MAD-3. The largest and most significant difference in this analysis was the detection of *Atherosclerosis Signaling* in skin lesions. The strength of the association between this pathway and the psoriatic phenotype is much stronger in the MAD-3 transcriptome (FDR<10^−5^) than in Suarez-Farinas+ (FDR<10^−2^). In addition, there were several IPA functions and pathways that were significant only in the MAD-3 (at FDR<0.1), including *Lymphoid Tissue Structure and Development*, and *Hypersensitivity Response* functions, as well as pathways such as *IL-2 Signaling, IL-17A Signaling in Fibroblasts, Granzyme B Signaling, MSP-RON Signaling Pathway,* and *Pathogenesis of Multiple Sclerosis.* Conversely numerous pathways identified uniquely by Suarez-Farinas+ in the bottom of the figure were not related to cytokine biology, so they appear to be of secondary importance within likely pathways of cytokine-drive pathogenesis. This finding supports the importance of the Meta-Analysis as an analytical approach to provide consensus on a molecular definition of psoriasis, as well as giving us new tools to explore the systemic associations that have been recently reported in psoriasis [22].

### Transcription Factors (TFs) Identified by the Meta-analysis

IPA also identified several transcription factors (TF) as being significantly activated or inhibited in this transcriptome (Table S5). Target molecules in the transcriptome predicted activation of TFs involved in interferon production, including IRF7, IRF1, IRF3, IRF5, STAT2, and T-box 21 (TBX21). This data, along with the above-mentioned interferon-associated canonical pathways, supports the involvement of interferons in psoriasis [26]. TBX21 is a Th1-specific TF that controls expression of IFNγ. Components of the NFκB pathway have been shown to be active in psoriasis [27], and NFκB and RELA TFs were both activated. High-mobility group box 1 protein (HMGB1), another activated TF, may be an important factor mediating the inflammatory response [28]. FOSL1 and Ap1 were both predicted to be activated TFs. FOSL1 can dimerize with Jun to form the Ap-1 complex, and the decreased activity of the pathway has been shown in a mouse model, with epidermal deletion of JunB/AP-1 causing psoriasis [29]. AP-1 TF is impaired in LS psoriasis skin [30]. FOXM1, a transcriptional activator involved in cell proliferation, was also activated, but a number of factors involved in cell cycling were inhibited, including RB1, SRF, CDKN2A, SMAD3, and SMARCB1. Transcription factors involved with myocardial differentiation and function were both activated (FOXO1) and inhibited (GATA4 and MYOCD) in this transcriptome. These observations support previous reports showing involvement of some of these TFs in psoriasis as described above, and provide opportunities to evaluate these newly identified TFs in the IPA of this MAD transcriptome.

### Integration Driven Discovery (IDD) “New” Genes

The MAD-3 identified a set of 64 genes which were not identified by any of the individual studies (Figure 2E) that we have termed Integration Driven Discovery (IDD) genes [17] (Table 2). The MAD-5 identified 11 IDD genes (Figure 2D), which were all found in the MAD-3 gene set of 64 except SERPINA5 and CYB5R2. In order to validate these results, 8 IDD genes were chosen due to their particular interest in psoriasis for RT-PCR confirmation using a new set of 9 patients with moderate-to-severe psoriasis. The RT-PCR results shown in Table 3 validated those genes as DEGs (p<0.05). The magnitude and direction of FCH for all genes under consideration were similar by PCR compared with the MAD-3 (Pearson correlation 0.98, p-value<0.001) Among these 64 IDD genes, 41% (26/64) were also detected by the above-mentioned pilot RNA-seq study (n = 3) [21] and the correlation between FCHs was 0.95 (p<10^−16^) (Table 2). Six of these genes (9.3%) were also detected by laser capture micro-dissection (LCM) [20] as DEGs in the Dermis (Table 2).

**Table 2:** Integration-Driven Discovery (IDD) genes in the MAD-3 transcriptome.

Symbol	Desc	LFC	FC	FDR	Rt-PCR	LFC LCM (D)^1^	LFC RNA-Seq	related gene- sets2	IPA^3^ Network
C18orf25	chromosome 18 open reading frame 25	1.01	2.02	<10^−5^				↑IFNγ	DD
HS3ST1	heparan sulfate (glucosamine) 3-O-sulfotransferase 1	1.26	2.40	<10^−5^				↑IFNγ, ↑inflammDC	DD
KIF15	kinesin family member 15	1.10	2.14	<10^−5^			0.70	↓IFNγ, ↓TNF	DD
HJURP	Holliday junction recognition protein	1.29	2.44	<10^−5^			1.17	↓IFNγ, ↓TNF	CD
LMNB1	lamin B1	1.08	2.11	<10^−5^		1.31*	0.66*	↓IFNγ, ↓TNF	CD
TDP1	tyrosyl-DNA phosphodiesterase 1	1.11	2.16	<10^−5^				↓IFNγ, ↓TNF	CD
TDO2	tryptophan 2,3-dioxygenase	1.37	2.59	<10^−5^		2.15*			CD
GNLY	granulysin	1.23	2.34	<10^−5^					CD
YOD1	YOD1 OTU deubiquinating enzyme 1 homolog (S. cerevisiae)	1.17	2.25	2.6×10^−3^			1.09		DD
CXCR6	chemokine (C-X-C motif) receptor 6	1.19	2.28	<10^−5^					DD
RPL27A	ribosomal protein L27a	1.11	2.16	2.0×10^−4^					DD
SH3GL3	SH3-domain GRB2-like 3	1.03	2.04	1.2×10^−3^					DD
CD28	CD28 molecule	1.02	2.03	7.0×10^−4^		2.59*	1.52	↑AD	IR
BAK1	BCL2-antagonist/killer 1	1.06	2.08	3.0×10^−4^			1.04	↑IFNγ	IR
PTPN22	protein tyrosine phosphatase, non-receptor type 22 (lymphoid)	1.23	2.35	2.0×10^−4^	*	1.54	1.35	↑inflammDC	IR
MUC4	mucin 4, cell surface associated	1.35	2.55	8.2×10^−3^					IR
LAIR2	leukocyte-associated immunoglobulin-like receptor 2	1.15	2.22	9.0×10^−4^					IR
CCL4	chemokine (C-C motif) ligand 4	1.09	2.12	2.0×10^−4^					IR
MBD1	methyl-CpG binding domain protein 1	1.08	2.12	<10^−5^					IR
WDR5	WD repeat domain 5	1.02	2.03	<10^−5^					IR
TSLP	thymic stromal lymphopoietin	1.02	2.03	<10^−5^					IR
WHSC1	Wolf-Hirschhorn syndrome candidate 1	1.04	2.05	<10^−5^				↓IFNγ	LM
TROAP	trophinin associated protein (tastin)	1.15	2.21	<10^−5^			1.04	↓IFNγ, ↓TNF	LM
EXO1	exonuclease 1	1.02	2.03	7.6×10^−3^				↓IFNγ, ↓TNF	LM
C13orf18	chromosome 13 open reading frame 18	1.12	2.18	<10^−5^		1.81			LM
ENTPD7	ectonucleoside triphosphate diphosphohydrolase 7	1.21	2.32	7.3×10^−3^					LM
GALE	UDP-galactose-4-epimerase	1.03	2.05	<10^−5^					LM
CASP5	caspase 5, apoptosis-related cysteine peptidase	1.02	2.03	3.4×10^−3^					LM
P2RX1	purinergic receptor P2X, ligand-gated ion channel, 1	−1.08	−2.11	5.0×10^−4^	*		−1.20		CD
RASSF6	Ras association (RalGDS/AF-6) domain family member 6	−1.00	−2.00	<10^−5^					CD
CEACAM7	carcinoembryonic antigen-related cell adhesion molecule 7	−1.09	−2.13	1.0×10^−2^					CD
SOX8	SRY (sex determining region Y)-box 8	−1.34	−2.53	<10^−5^					CD
ALDH1A2	aldehyde dehydrogenase 1 family, member A2	−1.24	−2.36	<10^−5^		−1.04*		DCs	DD
SLC27A2	solute carrier family 27 (fatty acid transporter), member 2	−1.25	−2.37	<10^−5^				↓AD	DD
PCDH20	protocadherin 20	−1.14	−2.20	<10^−5^			−1.83		DD
SRGAP1	SLIT-ROBO Rho GTPase activating protein 1	−1.00	−2.00	<10^−5^	*		−0.72		DD
TMPRSS11E	transmembrane protease, serine 11E	−1.49	−2.81	3.0×10^−4^	*				DD
MBP	myelin basic protein	−1.01	−2.01	<10^−5^				↑IFNγ	IR
FERMT2	fermitin family member 2	−1.17	−2.25	2.0×10^−3^				↑IFNγ	IR
MERTK	c-mer proto-oncogene tyrosine kinase	−1.16	−2.23	<10^−5^	*		−0.74	↓AD	IR
PPARG	peroxisome proliferator-activated receptor gamma	−1.14	−2.20	1.0×10^−4^	*			↓AD	IR
CFTR	cystic fibrosis transmembrane conductance regulator (ATP-binding cassette sub-family C, member 7)	−1.08	−2.11	1.6×10^−2^			−1.56		IR
MYOC	myocilin, trabecular meshwork inducible glucocorticoid response	−1.48	−2.78	<10^−5^			−1.46		IR
DCN	decorin	−1.13	−2.18	7.0×10^−4^			−0.59		IR
DACH1	dachshund homolog 1 (Drosophila)	−1.02	−2.03	<10^−5^					IR
CNTN4	contactin 4	−1.23	−2.35	<10^−5^			−1.53	↓AD	LM
PLEKHA6	pleckstrin homology domain containing, family A member 6	−1.06	−2.08	<10^−5^			−0.83	↓AD	LM
GLRB	glycine receptor, beta	−1.03	−2.04	<10^−5^				↓AD	LM
PECR	peroxisomal trans-2-enoyl-CoA reductase	−1.26	−2.39	<10^−5^				↓AD	LM
CNKSR2	connector enhancer of kinase suppressor of Ras 2	−1.47	−2.77	1.1×10^−3^				↓AD	LM
MEGF10	multiple EGF-like-domains 10	−1.05	−2.06	<10^−5^			−0.95		LM
BACH2	BTB and CNC homology 1, basic leucine zipper transcription factor 2	−1.18	−2.26	<10^−5^	*				LM
SLC28A3	solute carrier family 28 (sodium-coupled nucleoside transporter), member 3	1.02	2.03	5.0×10^−3^			1.07	↑IL-1	
SLCO4C1	solute carrier organic anion transporter family, member 4C1	−1.17	−2.25	7.5×10^−3^			−2.12	↓AD	
TOX3	TOX high mobility group box family member 3	−1.02	−2.02	<10^−5^				↓AD	
LRFN5	leucine rich repeat and fibronectin type III domain containing 5	−1.06	−2.08	1.0×10^−3^			−1.40		
FAM19A5	family with sequence similarity 19 (chemokine (C-C motif)-like), member A5	−1.04	−2.06	<10^−5^			−1.36		
LONRF2	LON peptidase N-terminal domain and ring finger 2	−1.26	−2.39	<10^−5^			−1.32		
C9orf152	chromosome 9 open reading frame 152	−1.46	−2.74	1.0×10^−4^			−1.28		
C4orf31	chromosome 4 open reading frame 31	−1.07	−2.09	<10^−5^			−1.07		
C1orf51	chromosome 1 open reading frame 51	−1.14	−2.20	<10^−5^			−0.69		
KIAA1239	KIAA1239	1.14	2.20	<10^−5^					
LOC375190	hypothetical protein LOC375190	−1.04	−2.06	<10^−5^					
BEX5	brain expressed, X-linked 5	−1.15	−2.22	<10^−5^					

1 Detected by LCM in the Dermis (no idd gene was detected in the Epidermis)

2 Gene-sets (defined by our group) with known role in psoriasis including keratinocytes’ response to IFNγ [9], [18], [31], TNF [9], [18], [31] and IL-1 [9], [18], [31], psoriasis inflammatory DC transcriptome [32] and AD transcriptome [33]

3 IPA Networks DD = Dermatological Disease and Conditions, CD = Cardiovascular System Development and Function, IR = cell-mediated Immune Response, LM = Lipid Metabolism

**Table 3:** RT-PCR validation on IDD genes.

		RT-PCR			Meta-analysis		
	Gene	LFC	p.value	FDR	LFC	p.value	FDR
1	P2RX1	−1.63	0.0128	0.0148	−1.08	0.0002	0.0005
2	TMPSS11E	−1.73	0.0122	0.0148	−1.23	0.0020	0.0024
3	BACH2	−1.38	0.0006	0.0024	−1.18	<0.0001	<0.0001
4	MERTK	−1.66	0.0004	0.0024	−1.01	<0.0001	<0.0001
5	PPARG	−1.25	0.0049	0.0098	−1.21	<0.0001	<0.0001
6	SRGAP1	−1.76	0.0015	0.004	−1.00	<0.0001	<0.0001
7	PTPN22	1.04	0.0130	0.0148	1.23	0.0001	0.0002
8	CYB5R2	0.73	0.0331	0.0331	1.01	<0.0001	<0.0001

Many of these IDD genes were found in gene-sets induced by IFNγ, TNF, and IL-1 cytokine- treated keratinocytes [9], [18], [31], as indicated in Table 2. Two up-regulated IDD genes (HS3ST-1 and PTPN22) were also present in the psoriasis inflammatory DC transcriptome [32], which contains DEGs between CD11c^+^CD11c^−^ DCs versus CD11c^+^CD1^+^ DCs FACS-sorted from psoriasis lesions. A number of these genes were also present in the atopic dermatitis transcriptome, with the same direction of dysregulation [33]. IPA analysis of these “new” IDD genes helped determine their relevance (Table 2). 17 of 64 genes belong to the *Cell-mediated Immune Response* network, 14 to *Lipid Metabolism*, 12 to *Dermatological Diseases and Conditions,* and 8 to *Cardiovascular Development and Function network*. 3 of these genes were found in the *Fatty Acid Metabolism* pathway (ALDH1A2, SCL27A2 and PCDH20). 2 genes were involved in *Type II Diabetes Mellitus Signaling* pathway (PPARG and SLC27A2), and there has been a SNP reported associated with type II diabetes for LONRF2. The *PPAR signaling pathway* was represented by 14/102 molecules in the MAD-3 (p = 0.08; FDR<0.3), and 2 IDD genes were associated with this pathway (PPARG and SLC27A2).

### Selection of Consistent Disease-classification Genes: A Genomic “Classifier” for Psoriasis

Biomarker discovery has become an important topic in biomedical research. There is a great interest in finding a small set of genes that best discriminate between diseased and healthy state, as these markers can lead to major biological insights regarding disease characteristics and diagnosis, as well as development of targeted therapeutics. Here we set out to identify the smallest set of genes that distinguish LS from NL skin samples across a heterogeneous cohort of psoriasis patients. With the MAD-5 transcriptome as a starting point, the Meta Threshold Gradient Directed Regularization (MTGDR) method proposed by Ma and Huang [16] was used to select genes most relevant to disease classification. This algorithm automatically establishes the minimal set of genes and a decision rule that classifies a given genomic profile of an unknown skin biopsy into LS or NL with a minimal error. Parameters of the model were estimated using the 386 available samples (training samples).

Although Ma and Huang [16] claimed that there is no limitation on the number of genes used as inputs for the MTGDR algorithm, when deciding their relevance, it is beneficial to assemble first the univariate selection of DEGs as starting point of MTGDR. In this way, a large amount of computing time can be saved with minimal difficulty in detecting potentially “true” biomarkers.

**Figure 5:**
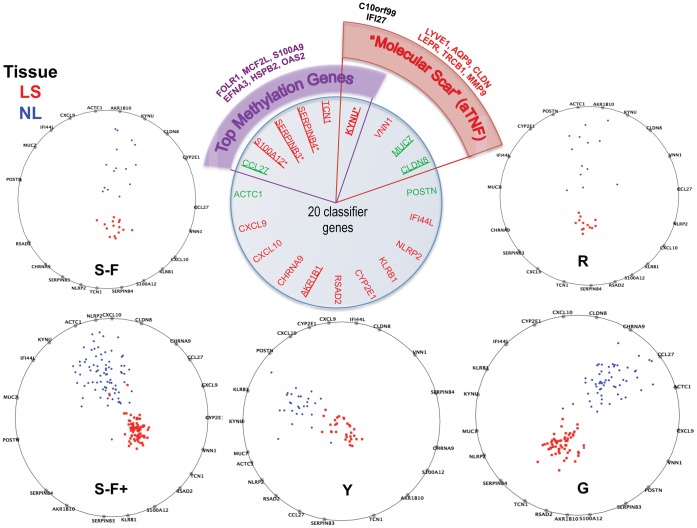
MAD classifier. Radviz plots showing how the 20 genes selected by MTGDR procedure separate the lesional (LS) and non-lesional (NL) samples apart in each study. Perfect separation between LS and NL samples can be seen in every study. S-F+: Suarez-Farinas 2012, hgu33plus2 chips; G: Gudhjonsson’2009; S-F: Suarez-Farinas’2010; R: Reischl’2007; Y: Yao’2008. Center insert shows biological relevance of these genes. Top 25 psoriasis genes in Table 1 are underline. 6 of these 20 genes have been identified as top methylation genes discriminating between psoriasis (LS) and healthy skin. 4/20 were identified as part of the Residual Disease Genomic Profile (RGDP) or “Molecular Scar”.

Using a 3 fold cross-validation, MTGDR’s tuning parameters (see equation 3 of Materials and Methods) were set as τ = 1 and k = 1298. With these tuning parameters and using all training samples, 20 genes were selected for the final model and used to construct the classifier. The performance among training samples was superb with only 2 samples (from Suarez-Farinas+ study) being misclassified (0.5%). The general classification error, estimated by a 5-fold cross-validation, was 1.3% and below 2% in all studies (Table S6). Those 20 genes and their corresponding estimates in each study are listed in Table S6. Here, a positive sign of the estimated coefficient indicates that up-regulation of this gene is positively associated with the outcome of interest, i.e., the odds of being LS skin. RadViz [34], a commonly used multidimensional visualization tool, was used to obtain a graphical representation of the disease-classification genes in a 2-dimensional space (Figure 5) for each study separately (see Materials and Methods for more details). RadViz graphs show a clear separation between LS and NL samples can be obtained with the 20 genes (biomarkers) whose biological relevance is addressed in the discussion.

Both misclassified samples were from Suarez-Farinas+ study, one NL sample misclassified as LS showed high K16 mRNA and abundant CD3^+^ T cell and CD11c^+^ dendritic cell infiltrates, similar to its LS pair. The second misclassified case was a LS sample from an Asian patient, and the cellular features of psoriasis were not very marked, for example epidermal thickness was similar to normal skin, and there were not many T cells or dendritic cells. This may be a specific type of psoriasis with a different “small plaque” morphology specific to this population [35].

The 20 genes in the classifier were reviewed for their presence in other studies [10], [36], as well as their biological relevance, as shown in the center top panel of Figure 5. The top 25 up- and down-regulated psoriasis DEGs from genes in Table 1 that are in the genomic classifier list are underlined. Six out of these 20 genes have recently been identified as top methylation genes discriminating between psoriasis (LS) and healthy skin [36]. 4/20 genes were identified as part of the Residual Disease Genomic Profile (RGDP) or more colloquially “molecular scar” after treatment with TNF blockade [10]. The methylation status of several of these classifier genes can also be considered as part of the molecular scar. Hence this classifier offers a novel tool for the molecular diagnosis of psoriasis, as well as for studying novel biology using model diseases. Furthermore, this meta-analysis provides a robust list of DEGs that can be mined for questions of pathogenicity, diagnosis, new therapeutics, and biomarkers, as shown above.

## Discussion

A statistically based meta-analytic approach systematically combines microarray studies from different patient populations and laboratories to provide a single estimate of the overall differential expression level for each gene. By accumulating results across studies, one can gain a more accurate representation of the population relationship than is provided by the individual study estimators; the statistical power is increased; the influence from any individual study is reduced. While individual studies generate variable sized lists of DEGs, the meta-analysis provides a more precise view of molecular definition of the disease, while simultaneously allowing for differences between studies. Of course, there are a number of issues associated with applying a meta-analysis in gene expression studies [12], [15]. For example, there are specific concerns regarding challenges with probes and probe sets, differential platforms being compared, and laboratory effects. To overcome these challenges, we carefully planned and conducted the meta-analysis from the very beginning of selecting the data sets. Using this approach and 5 microarray studies, we present a **M**eta-**A**nalysis **D**erived (MAD) transcriptome of psoriasis DEGs from a large sample size of 193 pairs of LS and NL skin biopsies. We believe that this list is more robust and consistent than can be obtained from a simple operation on (e.g., intersection or union) separate DEGs lists from individual studies.

Hopefully, other investigators will find this list of DEGs useful in defining a “core transcriptome” across a range of severity of psoriasis. The MAD-3 transcriptome was obtained from patients with “plaque-type”, “chronic” and “moderate to severe” psoriasis, while the 5 study also included patients with “mild to severe” disease, suggesting that these results represent the transcriptome from a range of severity with broad applicability across this disease. However, as the full details of all the patients in the contributing 5 studies were not readily available, prospective studies should be carried out on psoriasis of varying degrees of severity to confirm and extend these observations.

Analysis of this list of DEGs in IPA indicated that several well-known key cytokine pathways (e.g., IL-17 and IFNγ) and pathways of great importance such as *Role of IL-17A in Psoriasis, Atherosclerosis Signaling*, and *Fatty Acid Metabolism* were significantly represented in the DEGs list. The overall importance of IL-17 signaling in psoriasis is highlighted by several recent studies in which major improvements in psoriasis were seen when IL-17 antagonists were tested in clinical trials [37]–[39]. However, the largest difference detected in IPA profiles of the MAD-3 transcriptome concerned *Atherosclerosis Signaling* (Figure 4). This association is of particular interest in light of the well-established link between moderate-to-severe psoriasis and a significantly increased risk of cardiovascular disease [40]. Furthermore, it now appears that some factors that influence cardiovascular risk may be produced locally in inflamed psoriasis skin lesions and could become systemically available to increase risk of vascular or cardiac pathology [8], [22].

Not only is this MAD-3 list much larger than the intersection of DEGs of individual studies, it also identifies several ‘new’ IDD genes relevant to psoriasis (Table 2). Of the up-regulated IDD genes, many are of great interest. For example, PTPN22 is a psoriasis risk gene with polymorphisms associated with early onset psoriasis, and CYB5R2 is involved in fatty acid metabolism. There were also several interesting down-regulated IDD genes, including the serine protease TMPRSS11E, also called DECS1. TMPRSS11E correlates with normal keratinocyte differentiation [41], so reduction is consistent with the loss of normal keratinocyte differentiation that occurs in psoriasis. BACH2 has been implicated as a type 1 diabetes risk factor by GWAS [42], and may have a role in response to viral antigens [43]. MERTK may play a role in clearance of apoptotic cells by antigen-presenting cells [44], or may act as anti-inflammatory, potentially allowing reduction of MERTK unrestrained TLR activation [45]. Polymorphisms of PPARG have been seen in a cohort of psoriatic arthritis [46]. PPARβ/δ activation, which can be antagonistic to PPARγ, has been shown in psoriasis skin [47], and activation of PPARβ/δ contributed to a psoriasis-like mouse model of disease [48]. Expression levels of the above genes were all confirmed by RT-PCR.

Interestingly, we observed that 9 of the 20 classifier genes are among the top 25 up- and down-regulated genes listed in Table 1. Even with such a substantial overlap, a psoriasis expert may still question why many well-known up-regulated genes (e.g., DEFB4) were not in the list of “classifier” genes. A top DEG gene is not necessarily a good “classification” gene. DEG are genes whose average expression values differ across tissues/classes, while a classification algorithm aims to discriminate among different tissue/class using the smallest possible set of genes (classifier genes). How the association between a gene and tissue or class is measured differs correspondingly. While detecting DEGs, such association is measured univariately by using a moderated (paired) t-test, whereas in the MTGDR, the association of gene expression and tissue/class is modeled multivariately with all genes considered simultaneously as covariates in the logistic regression model. In this regard, MTGDR does two tasks simultaneously: building a classification rule and selecting the most relevant genes. Additionally top psoriasis DEGs are defined considering the paired structure of the data. A gene with a large between-patient variation may be a DEG (as defined by the paired t-test) but not necessarily ideal for prediction purposes.

Among classifier genes, TCN1 (transcobalamin 1/ HAPTOCORRIN) anchors (in Radviz plots) are consistently close to LS skin across different studies. This gene encodes a member of the vitamin B12-binding protein family cobalamin metabolic process, which is found in neutrophilic granules. TCN1 (Chr 1, LOD = 4.41) has shown linkage to serum insulin concentrations in impaired glucose tolerance [49]. TCN1 protein was also increased in synovium of rheumatoid arthritis [50], and was significantly associated with cholesterol levels or statin response [51], perhaps providing a predisposing link between both skin inflammation and high cholesterol.

Several genes in the psoriasis classifier, KYNU (up), MUC7 and CLDN8 (down), were part of the Etanercept “molecular scar” previously reported by our group [10]. The molecular scar represents a group of genes that are still expressed at the end of 12 weeks of successful treatment with etanercept, an anti-TNF agent used for psoriasis, at the time point where there was complete clinical resolution and no visible skin inflammation. KYNU, kynureninase, is an enzyme involved in the biosynthesis of NAD cofactors from tryptophan through the kynurenine pathway. Several genes in the classifier were also recently identified by Robertson *et al.* [36] as top genes harboring differential methylation sites in psoriasis versus normal skin, namely S100A12, SERPINB3, and KNYU. These investigators showed that patterns of DNA methylation of LS skin could help separate psoriatic LS from normal skin (with NL skin showing intermediate patterns of methylation). In this analysis, these three genes were in the top 10 most significant methylation sites, although there was an inverse correlation between DNA methylation and nearby gene expression for these genes. Hence KNYU may be a novel gene to evaluate in the future as a biomarker, both for its increased gene expression in LS skin as well as demethylation status. The presence of these genes in the classifier, which can be broadly considered a genomic predictor of disease, in clinically resolved psoriasis lesions, and as top sites harboring DNA methylation, may suggest their role as key genes in the molecular fingerprint of psoriasis. Further studies are warranted to determine their role and effects and future use as predictors of disease.

In conclusion, the meta-analysis produced a pool of consistent candidate genes for further investigation of psoriasis pathology, biomarker selection, and potential targeted treatment detection. In future work, these data may serve as a “gold standard” psoriasis transcriptome, since it has been carefully curated and modeled. Findings presented here can be further validated through RT-PCR or protein staining of important genes. It will be useful to examine the relationship between the top DEGs with clinical disease severity, to evaluate “new” genes in the transcriptome and their role in disease pathogenesis, and explore relationships between top DEGs, classifier genes, RDGP, and methylation. The transcriptome can also be used in the context of response to treatment, such as we have conducted in the past with etanercept treatment [6], [9] and ixekizumab [52]. The impact of the classifier genes can be studied alone, such as for TCN1, or they can be considered together as the molecular definition of psoriasis, which could aid in differential diagnosis. Specific new pathways identified by IPA provide opportunities for discover of disease pathogenesis and new therapeutic targets.

## Materials and Methods

Given the fact that only a careful application of meta-analysis can mitigate or overcome the variations across different studies, we chose the experiments on the same platform, and reanalyzed the data using the same preprocessing and analytic procedures. We followed the PRISMA statement guidelines, the corresponding checklist can be found in Table S7.

### The Experimental Data

We searched the NIH’s GEO (Gene expression omnibus) repository using *psoriasis* and *Affy chips on human* as keywords (before Oct 1^st^, 2009) identifying 8 potential experiments. One additional experiment was part of a collaboration with Janssen Research & Development, and has recently being released to GEO repository. We excluded 3 studies conducted on earlier Affymetrix HGU95 chips series (a-e and v2), while a fourth study was excluded because it was conducted on multiple outdated platforms. The inclusion of those studies would have severely limited the universe of genes to be analyzed and would had resulted in noisier data since the agreement with newer platforms is smaller.

Among five of the studies, 3 studies were performed on HGU133plus2 chips. The first study reported by Yao *et al.* [4], used 56 samples (28 LS and NL skin pairs) and found 1408 up-regulated and 1465 down-regulated probesets (974 and 853 genes respectively). The second was reported by Gudjonsson *et al.* [43] with a sample size of 116 (58 LS and NL pairs), and there was a set of 721 up-regulated and 364 down-regulated probesets (508 genes and 248 genes) identified. The third experiment [8] is a study with a total of 162 samples (81 LS and NL skin pairs) involved, and there were 2129 up-regulated probesets (1568 genes) and 2046 down-regulated probesets (1555 genes) reported. Also, a study with 15 LS and NL pairs on HGU133a2 chips published by Suarez-Farinas et al. [6] presented a list of 732 (579) up-regulated and 890 (703) down-regulated probesets (genes). In the last study, Reischl *et al.* [7] reported 179 DEGs using an experiment on HG-U133A chips with 13 patients. Table S1 summarizes the relevant information for the 5 studies used in the meta-analysis.

### Uniform Preprocessing of Raw Data for the Meta-analysis

The raw Affymetrix data (CEL files) of every study was downloaded from NIH’s GEO (Gene expression omnibus) repository and expression values were obtained using GCRMA algorithm [53] and normalization across samples was carried out using quantile normalization. As the first step of data filtering, only those probe sets that demonstrated a certain degree of variation across samples in each study were selected. Probe sets with SD below 0.1 were regarded as non-informative and eliminated. The set of common probe sets across studies was input for the further filtering by integrated correlation approach.

### Integrated Correlation and Gene-Coherence Score

The Integrated Correlation Coefficients (ICC) approach, introduced by [16], was used as the second step of data filtering. It defines a set of genes exhibiting coherent behavior across studies as explained in [54].

For each study s, x_g_ representing the expression profile for a gene *g,* and 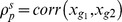 is the correlation for the pair of genes p = (g_1_,g_2_). The integrated correlation, defined as 
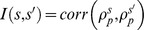
 quantifies the coherence between studies. If this expression is calculated considering only the pairs containing a specific gene g_k_, a measure of the gene-specific coherence between two studies is produced, which is 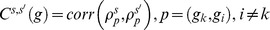. When more than two studies are involved, the average over all s and s’ is used as a Coherence Score for a gene g,
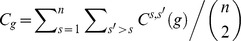



For each gene, a Coherence score was calculated and genes with scores bigger than the median were selected as a set of Coherent Genes. Confidence Intervals for the correlation scores were obtained by bootstrapping.

We evaluated how the results varied by the choice of cut-off in the filtering out of incoherent genes. For the 3 plus2-studies, using all genes or filtering out those on the first quartile (25%) of coherence score resulted in the identification of the same DEGs (data not shown). In the case of the 5 studies meta-analysis, the choice of 25% or 50% cut-offs rendered identical results (data not shown). Based on the above observations, it is reasonable to conclude that the implementation of filtering based on coherence scores ruled out mostly inconsistent but insignificant genes (random noises).

### Meta Analysis

The classic application of meta-analysis is to find a single outcome using published data where only the summary statistics are typically available. With microarray experiments, however, a more fortuitous situation of having the complete set of raw data available is commonly achievable. Thus, we took advantage of this feature and modeled the differences in expression values between LS and uninvolved (NL) skin pairs uniformly.

The general model in a meta-analysis setting is as follows. Let *Y*_ij_ represent the measured effect for study j (*j* = 1, …, J) for a specific gene *i*. We have,
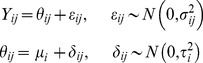
(1)where between-study variance *τ_i_* ^2^ represents the variability between studies, and it is usually estimated by the DerSimonian and Laird method [55]. And σ^2^ represents the within-study variance for the *i*th study. Both *Y*_ij_ and σ^2^ (called as summary statistics) are already known from previous analysis/study. *µ_i_* is regarded as the average measure of differential expression across all datasets/studies for this gene, which is the parameter of interest and may be estimated along with its a standard error (se) as: 
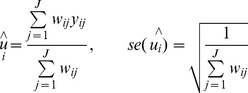
(2)where *w_ij_* equals to the inverse of the variance of *Y_ij_*. A question that must be addressed in meta-analysis is to specify if a fixed-effect model or a random-effects model is more appropriate for the data. This depends on the value of the between study variance; *τ_i_* ^2^ = 0 corresponds to a fixed model while *τ_i_* ^2^>0 corresponds to a random model. The hypothesis (Ho: *τ_i_* ^2^ = 0 versus Ha: *τ_i_* ^2^>0) would be tested using the Cochran’s Q statistic that follows a χ^2^_n-1_ distribution under the null hypothesis [17].

### Model Fitting

For each study, a moderated paired t-test was used to analyze differences (on the log_2_ scale) among LS and NL psoriatic samples. The summary statistics (*Y_ij_* and σ^2^ in Equation 1) were recorded and would be the input in the meta-analysis. Then, the overall fold changes (LFC) between LS and NL skin on the log_2_ scale (i.e., the parameters of interest in this analysis) and their corresponding standard errors were calculated using Equation 2. The resulting adjusted p-values using Benjamini and Hochberg procedure, which control for false discovery rate (FDR), are used to decide the statistical significance of genes along with LFC.

### Disease-classification Genes

The Meta Threshold Gradient Directed Regularization (MTGDR) method proposed by Ma and Huang [16] was used to select genes (also referred to as biomarkers) which may distinguish LS and NL skin samples. MTGDR is an extension of the Threshold Gradient Directed Regularization (TGDR) [56], to the case where several studies are combined. For each study m, the independent variables Y_m_ - defined as the binary indicators of group membership (1 denotes LS skin and 0 for NL skin) - is modeled through a logistic regression with the expression values for all genes (represented by matrix matrix X_m_) as a covariates. MTGDR assumes that the regression coefficients of the logistic regression of the TGDR model may be different across studies but the sets of genes with nonzero coefficients (i.e., the classifier/biomarker genes) are the same across studies.

In microarray studies where more than thousands of genes are surveyed, only a small number of genes are actually associated with the outcome of interest. MTGDR tries to select such genes (corresponding to those with nonzero coefficients on the logistic regression) and estimates the corresponding coefficients simultaneously by maximum likelihood. The algorithm starts with initial values for the regression coefficients equal zero and in each iteration, updates only the coefficients associated with genes with large meta-gradients (defined by the sum of the gradient across different experiments). Which and how many genes are updated in each iteration are determined by k (the number of iterations) and the tuning parameter τ. A value of τ = 1 indicates that only the gene with largest meta-gradient is updated whereas if τ = 0, all genes will be updated. With a large τ (close to 1) and a finite k, only a small number of genes will have nonzero coefficients. For the detailed descriptions on MTGDR, see Ma and Huang [16].

Tuning parameters τ and k were jointly determined by a 3-fold cross-validation. Samples from each study were randomly divided into 3 subsets and cross-validation was carried out by running MTGDR for a set of possible values for τ (range from 0–1) and number of iterations (k) up to 5000. Optimal parameters were selected as those that maximized the log-likelihood. The limit of 3-fold cross-validation was due to the small size in one study. To evaluate the performance of the final classifier, we considered 5-fold cross-validation. Samples in each study were randomly divided into 5 parts, 4 folds were used to run MTGDR, and then the resulting estimates were used to make prediction on the removed one fold. This procedure was repeated 5 times to produce class prediction on all samples, and the prediction error was computed. Prediction errors generated by 10 and 20-fold cross-validation produced similar results.

### Radviz Plot

Radial Coordinate Visualization (Radviz) [8] is a non-linear visualization technique that can display data on three or more attributes in a 2-dimensional projection. The visualized attributes (e.g., genes) are presented as anchor points spaced around the perimeter of a circle. Samples are shown as points inside the circle, with their positions determined by a metaphor from physics: each point is held in place with springs that are attached at the other end to the attribute anchors. The stiffness of each spring is proportional to the value of the corresponding attribute and the point ends up at the position where the spring forces are in equilibrium. Data instances that are close to a set of feature anchors have higher values for these features than for the others. A unique Radviz plot including all 5 studies was not possible in practice since the odds of being LS skin differs among individual studies as shown in Table S4.

### RT-PCR

The gene expression data for certain genes of interest were confirmed by RT-PCR in NL and LS biopsies from 9 patients with moderate-to-severe psoriasis. All patients gave written informed consent, and research was approved by the Rockefeller University Institutional Review Board. RNA was extracted from skin samples frozen in liquid nitrogen using the RNeasy Mini Kit (Qiagen, Valencia, CA). DNA was removed by on-column DNase digestion by the Qiagen RNase-free DNase Set. The primers for TMPSS11E, BACH2, MERTK, PPARG, RASSF6, SRGAP1, BAK1, PTPN22, CYB5R2 (Hs01070171_m1, Hs00222364_m1, Hs01031973_m1, Hs01115513_m1, Hs00698249_m1, Hs00381035_m1, Hs00832876_g1, Hs00249262_m1, Hs00212055_m1, respectively) were obtained from Applied Biosystems (Foster City, CA). Data was normalized against a housekeeper gene, human acidic ribosomal protein. Paired t-tests were conducted on the log2 transformed expression values for each gene, and corresponding p-values were reported.

### Ingenuity Pathway Analysis

IPA software (www.ingenuity.com) was used to examine the data in the context of known biological response and regulatory networks as well as other higher-order response pathways. IPA uses a Fisher’s exact test to determine the probability of each biological function or disease assigned to MAD transcriptome by chance. In the functional network analysis, genes are grouped in networks with connections representing known biological relationships, supported by a published reference. IPA was also used to predict which transcription factors could be responsible for gene expression and whether those transcription factors are activated or inhibited.

### Statistical Language and Packages

The statistical analysis was carried out in the R language version 2.12 (www.r-project.org), and packages were from the Bioconductor project (www.bioconductor.org). The RadViz plots were made in the Orange software (www.orange.biolab.si).

## Supporting Information

Figure S1 A. Model selection. QQ plot showing the comparison of sample quantiles of Cochran’s Q against the quantiles of χ^2^_n−1_distribution (the theoretic distribution under the null hypothesis, n is the number of studies), the substantial deviation indicates a random effect meta-analysis model is preferred. B. QQ plot showing the comparison of the standardized overall effect estimates using the random effect meta-analysis model with a standard normal distribution, which indicates that those estimates do not deviate too far from normality.
(PDF)

Table S1 **Description and summary of the analytic methods used in the original studies.**
(PDF)

Table S2 MAD-5 psoriasis transcriptome. DEGs identified by the 5-study meta-analysis.
(XLS)

Table S3 MAD-3 psoriasis transcriptome. DEGs identified by the 3-study meta-analysis.
(XLS)

Table S4 Significant canonical pathways represented in MAD-3 transcriptome.
(XLS)

Table S5 Transcription Factors predicted to be Activated/Inhibited by IPA analysis.
(PDF)

Table S6 The 20 “classification” genes selected by MTGDR procedure. A. Estimated coefficients for each gene in each study. B. Misclassification error rates in each study and an overall error rate using a 5-fold cross-validation.
(PDF)

Table S7 The PRIMA (Preferred Reporting Items for Systematic Reviews and Meta-Analyses) checklist.
(DOC)
