# Supplementary figures and images for: Meta-Analysis Derived (MAD) Transcriptome of Psoriasis Defines the “Core” Pathogenesis of Disease
Source: PLoS One. 2012 Sep 5;7(9):e44274. doi: 10.1371/journal.pone.0044274 (PMC3434204; doi:10.1371/journal.pone.0044274)

Supplementary Figure 1

A

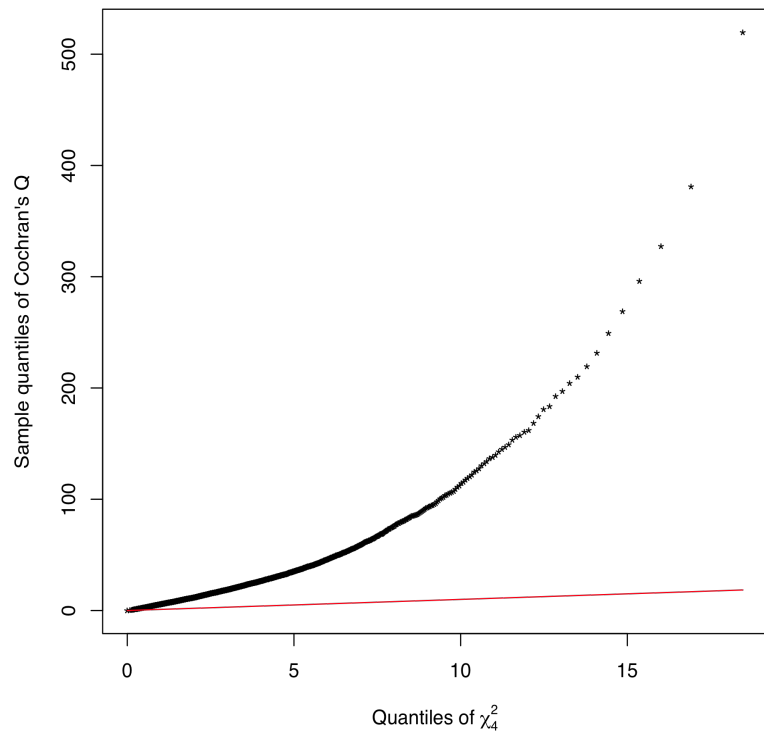

B

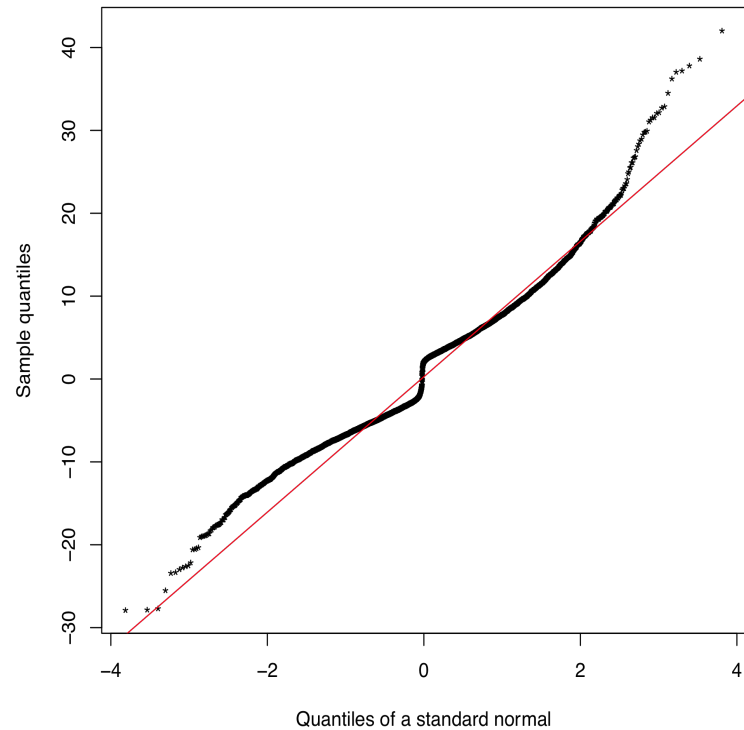

Supplement: Figure S1 — A. Model selection. QQ plot showing the comparison of sample quantiles of Cochran’s Q against the quantiles of χ2 n−1distribution (the theoretic distribution under the null hypothesis, n is the number of studies), the substantial deviation indicates a random effect meta-analysis model is preferred. B. QQ plot showing the comparison of the standardized overall effect estimates using the random effect meta-analysis model with a standard normal distribution, which indicates that those estimates do not deviate too far from normality. (PDF) [file pone.0044274.s001.pdf]
