# Supplementary material for: Meta-Analysis Derived (MAD) Transcriptome of Psoriasis Defines the “Core” Pathogenesis of Disease
Source: PLoS One. 2012 Sep 5;7(9):e44274. doi: 10.1371/journal.pone.0044274 (PMC3434204; doi:10.1371/journal.pone.0044274)
Supplement: Table S1 — Description and summary of the analytic methods used in the original studies. (PDF) [file pone.0044274.s002.pdf]

	Reischl	Suarez-Farinas	Yao	Gudjonsson	Suarez-Farinas +
Platform	hgu133a	hgu133a2	hgu133plus2	hgu133plus2	hgu133plus2
# of Chips	26	30	52	116	162
# of Patients	13	15	26	58	81
GEO Accession #	GSE6710	GSE11903	GSE14905	GSE13355	
Plaque Psoriasis	Mild to severe	Moderate to severe	Plaque type	Chronic	Moderate to severe
Preprocessing Algorithm	MAS 5.0	GCRMA	GCRMA (ArrayAssist Lite software)	RMA (extra normalization for gender/batch effects)	GCRMA
Filtering	SD>0.1 FC>2	SD>0.1	Not mentioned (NA)	Not mentioned (NA)	SD>0.1
Statistical method	Paired t-test Paired Wilcoxon test	Moderated paired t-test	SAM (R package's samr)	Paired T-test	Moderated paired t-test
Multiple Hypothesis' Correction	None	Benjamini-Hochberg	Q-value were calculated	Bonferroni correction	
Cutoffs: FDR / p-value Fold Change (FC)	p<0.00001 (t-test) p<0.001 (Wilcoxon)	FDR<0.05 FCH>2	Q-value <0.05 FCH>2	FDR<0.05 FCH>2	FDR<0.05 FCH>2
# Up-regulated probe-sets (genes)	203 (179)[1]	732 (579)	1408 (974)	721 (508)	2129(1568)
# of Down-regulated probe-sets (genes)		890 (702)	1465 (851)	364 (247)	2046(1145)

[1] Only the total number of significantly expressed probe sets (genes) was given in the original paper.
