# Supplementary material for: Meta-Analysis Derived (MAD) Transcriptome of Psoriasis Defines the “Core” Pathogenesis of Disease
Source: PLoS One. 2012 Sep 5;7(9):e44274. doi: 10.1371/journal.pone.0044274 (PMC3434204; doi:10.1371/journal.pone.0044274)
Supplement: Table S5 — Transcription Factors predicted to be Activated/Inhibited by IPA analysis. (PDF) [file pone.0044274.s006.pdf]

TR	Type	LR-Meta	PAS-Meta	Z-score Meta	p-Meta	LR-S-F+	PAS-S-F+	Z-score S-F+	p S-F+	Target Meta	Target S-F+
CDKN2A	TR		Inh	-2.958	2.3E-06		Inh	-3.19	1.37E-05	ASF1B, ATAD2, AURKB, BCL2, BIRC5, BLM, CCL2, CCNA2, CCND1, CCNE1, CDCA5, CDK1, CENPK, DHFR, FBXO5, FEN1, FST, KCNK1, MCM4, MELK, MMP1 (includes EG:300339), PPFIBP1, RAB27A, RAD51AP1, RRM2, TFDP1, TK1, TMPO, ZNF655	ASF1B, ATAD2, BARD1, BCL2, BIRC5, BLM, CCL2, CCNA2, CCNE1, CDCA5, CDK1, CENPK, CITED2, DHFR, E2F2, FANCA, FBXO5, FEN1, FST, GPRC5A, KCNK1, MELK, MMP1 (includes EG:300339), NR3C1, PPFIBP1, RAB27A, RAD51AP1, RRM2, TCF19, TFDP1, TK1, TMPO, TP63, ZNF655
MYOCD	TR	-3.33	Inh	-2.842	0.015	-3.330	Inh	-2.50	3.30E-01	ACTA1, ACTA2, CNN1 (includes EG:1264), FOS, MYH11, TAGLN, TNNT2	ACTA2, CACNA1D, MYH11, TAGLN, TNNT2
Nr1h	group		Inh	-2.504	0.0221		Inh	0.00		APOC1, APOD, APOE, CCL5, CXCL1, IL8, LPL	APOC1, APOD, APOE, CCL5, CXCL1, IL8, LPL
RB1	TR		Inh	-2.505	9.6E-07		Inh	-3.18	1.51E-05	ASF1B, ATAD2, BCL2, BIRC5, BLM, CCNA2, CCNB1, CCND1, CCNE1, CDC6 (includes EG:23834), CDCA5, CDK1, CENPK, CYP27B1, DHFR, F3, FBXO5, FEN1, FOS, FST, ID1, IVL, KCNK1, KIT, LACTB, LCK, MCM10 (includes EG:307126), MCM4, MCM5, MELK, PPFIBP1, RAB27A, RAD51, RAD51AP1, RRM2, SOX7, TFDP1, TMPO, TYMS, ZNF655	ASF1B, ATAD2, BARD1, BCL2, BIRC5, BLM, CASP4, CBX5, CCNA2, CCNB1, CCNE1, CDCA5, CDK1, CENPK, CITED2, CYP27B1, DHFR, F3, FANCA, FBXO5, FEN1, FST, GPRC5A, ID1, IVL, KCNK1, KRT4, LCK, MCM10 (includes EG:307126), MCM2, MCM5, MELK, MYC, PPFIBP1, PPT2 (includes EG:100126133), RAB27A, RAD51, RAD51AP1, RBL2 (includes EG:100331892), RRM2, SOX7, TCF19, TFDP1, TMPO, TYMS, ZNF655
Smad2/3-Smad4	complex		Inh	-2.329	0.0564		Inh	-2.25	4.91E-02	BCL2, COL1A2, IGFBP5, ITGB5	BCL2, COL1A2, IGFBP5, ITGB5, MYC
SMAD3	TR		Inh	-2.978	0.00289		Inh	-2.73	3.38E-02	ACTA2, ASPN, COL1A2, FBLN1, FOS, FST, GZMA, GZMB, HMOX1, ID1, ITGB5, MMP1 (includes EG:300339), POR, RHOB, TIMP3, TPM1 (includes EG:22003), TPM2	ASPN, COL1A2, FBLN1, FST, GLI2, GZMA, GZMB, HMOX1, ID1, ITGB5, MMP1 (includes EG:300339), MYC, POR, TGFB2, TIMP3, TPM1 (includes EG:22003), TPM2
SMARCB1	TR		Inh	-3.136	4.4E-10		Inh	-3.04	2.76E-05	ADD3, CCNA2, CCND1, CDC6 (includes EG:23834), CDK1, CDT1, CENPA, CENPE, CXCR4, DACH1, DHFR, F11R, FABP4, FZD7, HBEGF, KIF11, KIF23, MCM10 (includes EG:307126), MCM4, MCM5, MMP1 (includes EG:300339), MX1, OAS1, OAS3, POSTN, PPARG, PTN, RAD51AP1, SGCG, SMC4, TFDP1	ACTR2, ADD3, CCNA2, CDK1, CDT1, CENPA, CENPE, DHFR, DOCK4, F11R, FABP4, FZD7, HBEGF, KIF11, KIF23, MCM10 (includes EG:307126), MCM2, MCM5, MMP1 (includes EG:300339), MX1, OAS1, OAS3, POSTN, PTN, RAD51AP1, SGCG, SMC4, TFDP1
MLL2	TR		Inh	-2.163	0.0231			-1.94	3.90E-04	CRIP1, CRIP2, FHL1 (includes EG:14199), PCDH7, PPP2R2B, TNNT2	CRIP1, CRIP2, CSPG4, CTSD, FABP3, FHL1 (includes EG:14199), NPR3 (includes EG:18162), PCDH7, PPP2R2B, TNNT2
SRF	TR		Inh	-2.571	0.0008			-1.29	6.90E-03	ACTA1, ACTC1, ACTG2, AKAP12, ARHGAP20, BCL2, CALD1, CNN1 (includes EG:1264), CYLD, DMD, FOS, FOSL1, LMCD1, MCL1, MGST1, MYH11, MYLK, RAI2, TAGLN, TNNT2	ACTC1, ACTG2, BCL2, CYLD, DUSP5, FOSL1, MCL1, MYH11, MYLK, TAGLN, TNNT2
GATA4	TR		Inh	-2.576	0.048			0.00		ACTA1, ACTC1, ACTG2, GATA3, MUC4, TAGLN	
FOXO3	TR			-1.143	1.3E-05		Inh	-2.63	1.37E-06	AR, BIRC5, CCNB1, CCND1, EIF4EBP1, FBXO32, FOXM1, FOXO1 (includes EG:2308), GRB14, HMGCS2, IL8, IRS2, LCN2, MKI67, MMP9, MXD1, OVOL1, PLAU, PPARGC1A, SOD2, TNFSF10, TXNIP, UBE2C	AR, BIRC5, CAT, CCNB1, CCNG2, CFLAR, CPT1A, EIF4EBP1, FBXO32, FOXM1, FOXO1 (includes EG:2308), GRB14, HMGCS2, IL8, IRS2, LCN2, MKI67, MMP9, MXD1, MYC, OVOL1, PLAU, PPARGC1A, RBL2 (includes EG:100331892), SLC7A1, SMAD4, SOD2, TGFB2, TXNIP, UBE2C
WT1	TR			-1.837	0.0474		Inh	-2.02	2.29E-01	AREG/AREGB, BAK1, BCL2, CCND1, CCNE1, EREG, HBEGF, MMP9, NTRK2	AREG/AREGB, BCL2, CCNE1, EREG, HBEGF, IGF2, MMP9, MYC, NTRK2
Ap1	complex		Act	2.046	7.1E-05		Act	2.19	1.42E-02	BAK1, BCL2A1, CCL2, CCL4, CCNA2, CCND1, F3, FABP4, FOS, FOSL1, GZMB, HMOX1, IL17D, IL1B, IL8, KRT18, KRT6A, MMP1 (includes EG:300339), MMP9, PLAU, PRDM1, SELE (includes EG:20339), SLC8A1	BCL2A1, CCL2, CCNA2, DEFB103A/DEFB103B, F3, FABP4, FOSL1, GZMB, HMOX1, IL8, KCNN4, KRT18, KRT6A, MMP1 (includes EG:300339), MMP9, MYC, NR3C1, PLAU, PRDM1, SELE (includes EG:20339), SLC8A1
EZH2	TR		Act	2.659	4.4E-05		Act	2.91	3.46E-03	ALOX5AP, BIRC3, C15orf48, CA6, CCND1, CCNE2, CLEC3B, CXCL1, CXCL10, CXCL11, CXCL2, CYP1B1, FBLN1, FHOD3, FRZB, GPR68, IKZF1, IL17A, IL8, KIAA1199, KRT6B, LCN2, LTB, LYPD6B, MPZL2, NCOA7, PCDH20, PPP2R2B, RBM5, SERPINA1, TBX3	BIRC3, C15orf48, CA6, CCNE2, CHRM3, CLEC3B, CSNK1A1, CXCL1, CXCL10, CXCL2, CYP1B1, FBLN1, FHOD3, FRZB, GPR68, HSD11B2, IKZF1, IL8, KIAA1199, KRT6B, LCN2, LYPD6B, MMP7, MPZL2, MYC, NCOA7, PPP2R2B, RBM5, SAA1, SERPINA1, SLC1A3, TRIM38
FOXM1	TR	4.91	Act	2.539	1.3E-08	4.910	Act	3.19	5.08E-05	AURKB, BIRC5, CCNA2, CCNB1, CCNB2, CCND1, CCNE2, CDC20, CDC25B, CDK1, CDKN3, CENPA, CENPF, FOXM1, KIF20A, MMP9, NEK2, PRC1 (includes EG:233406)	BIRC5, CCNA2, CCNB1, CCNB2, CCNE2, CDC20, CDK1, CDKN3, CENPA, CENPF, FOXM1, KIF20A, MMP9, MYC, NEK2, PRC1 (includes EG:233406)
FOXO1 (includes EG:2308)	TR	-2.88	Act	3.044	3.4E-11	-2.880	Act	2.43	5.34E-08	ALAS1, ANLN, ASPM, BCL2, BCL2A1, BID, BIRC3, BIRC5, BRIP1, CCNB1, CCNB2, CCND1, CDK1, CENPF, DEPDC1, DLGAP5, EIF4EBP1, FBXO32, FOXO1 (includes EG:2308), HUS1, IL17A, IRS2, KIF11, MCM5, MMP1 (includes EG:300339), NCAPG, NEK2, NUSAP1, OVOL1, PCK1 (includes EG:18534), PPARG, PPARGC1A, PRC1 (includes EG:233406), SOD2, SPC25 (includes EG:100144563), TNFSF10, TXNIP	ALAS1, ASPM, BCL2, BCL2A1, BCL2L13, BID, BIRC3, BIRC5, BRIP1, CCNB1, CCNB2, CCNG2, CDK1, CENPF, CFLAR, CITED2, DEPDC1, DLGAP5, EIF4EBP1, FBXO32, FOXO1 (includes EG:2308), HUS1, IRS2, KIF11, MCM5, MMP1 (includes EG:300339), MYC, NCAPG, NEK2, NUSAP1, OVOL1, PPARGC1A, PRC1 (includes EG:233406), RPS6KA3, SMAD4, SOD2, SPC25 (includes EG:100144563), TXNIP
HMGB1	TR		Act	2.759	0.00188		Act	2.12	5.49E-02	CCL2, CCL20, CCL4, CD83, IFIT1, IL1B, IL8, MMP1 (includes EG:300339), MMP9, SELE (includes EG:20339), TLR2	CCL2, CCL20, CD83, IFIT1, IL8, MIA, MMP1 (includes EG:300339), MMP9, SELE (includes EG:20339), TLR2
IRF1 (includes EG:16362)	TR	5.93	Act	3.029	2.5E-13	5.930	Act	2.82	4.18E-06	BAK1, BCL2, CASP1, CCL19, CCL5, CEACAM1 (includes others), CXCL10, IDO1, IFI35, IFIH1, IFIT1, IFIT3, IL1B, IL8, IRF1 (includes EG:16362), IRF7, IRF9, ISG15, MMP9, MX1, OAS1, OAS2, PLA2G16, PSMB10, PSME2, RSAD2, SLPI, SOCS1, STAT1, STAT3, TAP2, TNFSF10, TRIM22	BCL2, CASP1, CEACAM1 (includes others), CXCL10, IFI35, IFIH1, IFIT1, IFIT3, IFIT5, IL8, IRF1 (includes EG:16362), IRF7, IRF9, ISG15, MMP9, MX1, OAS1, OAS2, PLA2G16, PSMB10, RSAD2, SLPI, SOCS1, STAT1, STAT3, TAP2, TRIM22
IRF3	TR		Act	2.861	6.8E-07		Act	2.50	2.61E-06	ARG2 (includes EG:11847), BIRC3, CCL19, CCL2, CCL5, CXCL1, CXCL10, FST, FUT2, GBP1, IFI44, IFI6, IFIT1, IFIT3, IL8, IRF7, ISG15, ISG20, MARCH6, MMP9, PNP, RSAD2, TIMP3, TMPO, TSLP	AHNAK, ARG2 (includes EG:11847), BIRC3, CCL2, CCL5, CDH11, CXCL1, CXCL10, FGF1, FST, FUT2, GBP1, IFI44, IFI6, IFIT1, IFIT3, IL8, IRF7, ISG15, ISG20, MARCH6, MMP9, NR3C1, PMAIP1, PNP, RSAD2, SORL1, TIMP3, TMPO
IRF5	TR		Act	2.359	1.1E-07		Act	2.06	3.19E-03	BAK1, CCL19, CCL4, CXCL11, IFI44, IFIT1, ISG15, NAMPT, OAS1, PLSCR1, PRKRA, RSAD2, SP110, TMPO, TNFSF10	IFI44, IFIT1, ISG15, NAMPT, OAS1, PLSCR1, PMAIP1, PRKRA, RSAD2, SP110, TMPO

IRF7	TR	7.11	Act	5.61	4.8E-20	7.110	Act	5.87	1.71E-13	APOBEC3G, CARD16, CCL19, CCL5, CXCL10, DDX58, GBP1, GBP3, GBP5, HERC5, IDO1, IFI35, IFI44, IFI44L, IFI6, IFIH1, IFIT1, IFIT3, IRF1 (includes EG:16362), IRF8, IRF9, ISG15, ISG20, MCL1, MICB, MX1, MX2, NAMPT, NMI, OAS1, OAS2, OAS3, OASL, PLSCR1, PSMB10, PSME2, RSAD2, RTP4, S100A8, SOCS1, STAT1, TAP2, TMPO, TNFSF10, TRIM22, UBE2L6, USP18, XAF1, ZC3HAV1	BCL2L13, CARD16, CASP4, CCL5, CXCL10, DDX58, GBP1, GBP3, GBP5, HERC5, IFI35, IFI44, IFI44L, IFI6, IFIH1, IFIT1, IFIT3, IRF1 (includes EG:16362), IRF8, IRF9, ISG15, ISG20, MCL1, MICB, MX1, MX2, NAMPT, NMI, OAS1, OAS2, OAS3, OASL, PLSCR1, PMAIP1, PSMB10, RSAD2, RTP4, S100A8, SOCS1, STAT1, TAP2, TMPO, TRIM21, TRIM22, UBE2L6, USP18, XAF1, ZC3HAV1
NFkB (complex)	complex		Act	4.818	2.2E-14		Act	5.37	1.47E-05	BAK1, BCL2, BCL2A1, BCL3, BIRC3, BIRC5, CCL2, CCL20, CCL22, CCL4, CCL5, CCL8, CCNB1, CCNB2, CCND1, CD274, CD83, CDC25B, CEBPD, CFTR, COL1A2, CXCL1, CXCL10, CXCL11, CXCL2, CXCL9, CXCR4, CYLD, DEFB4A/DEFB4B, E2F7, EHF, ELF3, ELL2, EPCAM, F3, FOS, FST, GATA3, GCH1, HERC5, HMOX1, IKBKE, IL17A, IL19, IL1B, IL1RN, IL32, IL7R, IL8, IRF1 (includes EG:16362), IRF7, ITGAL, KIT, KRT17, KRT6A, LCN2, LSP1 (includes EG:16985), LTB, MCL1, MFHAS1, MMP1 (includes EG:300339), MMP9, MYLK, NAMPT, NCF2, NCOA7, NFKBIZ, NOS1, PLAU, PPARG, PPIF, REL, SDC4, SELE (includes EG:20339), SERPINA3, SLIT2, SOAT1, SOCS1, SOCS3, SOD2, TAP2, TLR2, TNFSF10, TSLP, WNT5A, ZC3H12A	ABCG1, BCL2, BCL2A1, BCL3, BIRC3, BIRC5, CCL2, CCL20, CCL22, CCL5, CCL8, CCNB1, CCNB2, CD274, CD83, CEBPD, CFB, CFLAR, COL1A2, CSNK2A1, CXCL1, CXCL10, CXCL2, CXCL9, CYLD, DEFB103A/DEFB103B, DEFB4A/DEFB4B, E2F7, EHF, ELF3, ELL2, EPCAM, F3, FGF1, FGF2, FST, GLI2, HERC5, HMOX1, IKBKE, IL19, IL1RN, IL7R, IL8, IRF1 (includes EG:16362), IRF7, ITGAL, KRT17, KRT6A, LCN2, LSP1 (includes EG:16985), MCL1, MFHAS1, MMP1 (includes EG:300339), MMP9, MTSS1, MYC, MYLK, NCF2, NCOA7, PECAM1, PLAU, PMAIP1, PPIF, SAA1, SELE (includes EG:20339), SERPINA3, SLC2A5, SLC7A1, SLIT2, SOAT1, SOCS1, SOCS3, SOD2, TACR1, TAP2, TLR2, TNFRSF4, WNT5A, ZC3H12A
RELA	TR		Act	3.72	1.2E-08		Act	2.57	9.31E-05	BCL2, BCL2A1, BCL3, BIRC3, CCL19, CCL2, CCL20, CCL5, CCND1, CFTR, COL1A2, CTSB, CXCL1, CXCL10, CXCL11, CXCL2, CXCL9, CXCR4, DEFB4A/DEFB4B, EHF, ELF3, F3, IKBKE, IL1B, IL1RN, IL20, IL32, IL7R, IL8, IRF1 (includes EG:16362), KIT, KRT15, LTB, MMP1 (includes EG:300339), MMP9, MUC1, NAMPT, NOD2, PI3, PLAU, PPARG, PSMB10, SDC4, SELE (includes EG:20339), SOD2, TAP2, TLR2, TSLP	BCL2, BCL2A1, BCL3, BIRC3, CAV1, CCL2, CCL20, CCL5, COL1A2, CTSB, CXCL1, CXCL10, CXCL2, CXCL9, DEFB4A/DEFB4B, EHF, ELF3, F3, FGF2, GLI2, IKBKE, IL1RN, IL7R, IL8, IRF1 (includes EG:16362), KRT15, MIA, MMP1 (includes EG:300339), MMP9, MUC1, MYC, NF2, NFKBIB, NOD2, PECAM1, PI3, PLAU, PLD1, PSMB10, SELE (includes EG:20339), SELP, SLC2A5, SOD2, TACR1, TAP2, TLR2, TNFRSF10A, TNFRSF4
STAT2	TR		Act	2.377	1.3E-11		Act	2.36	5.24E-08	BCL2, CCL19, CXCL10, GBP1, IFI27, IFI35, IFI6, IFIT1, IFIT3, IL8, IRF1 (includes EG:16362), IRF9, ISG15, MX1, OAS1, TNFSF10	BCL2, CXCL10, GBP1, IFI27, IFI35, IFI6, IFIT1, IFIT3, IL8, IRF1 (includes EG:16362), IRF9, ISG15, MX1, OAS1
TBX21	TR		Act	2.132	0.00174		Act	0.00		GATA3, GZMB, IL12RB1, IL12RB2, PRF1, RORC, SELPLG	GATA3, GZMB, IL12RB1, IL12RB2, PRF1, RORC, SELPLG
FOSL1	TR	**18.2**	**Act**	**2.1**	**0.00244**	18.200		1.83	5.22E-02	**CYP2J2, FOS, IL8, IVL, MMP9, SPRR1B, THBD**	CYP2J2, IL8, IVL, MMP9, SPRR1B, THBD
E2F1	TR			1.763	7.1E-06		**Act**	2.28	5.87E-06	AR, AURKA, AURKB, BAK1, BCL2, BID, BIRC5, CALD1, CCNA2, CCNB1, CCND1, CCNE1, CCNE2, CDC6 (includes EG:23834), CDK1, CHEK1, CRYAB, CTNNBIP1, CTSB, CYP27B1, DHFR, ECT2, FBXO5, FEN1, FHL1 (includes EG:14199), FOXM1, GINS1, H19, HELLS, HN1, JMY, KIAA0101, LACTB, LCK, MAD2L1, MCL1, MCM10 (includes EG:307126), MCM4, MCM5, MMP1 (includes EG:300339), NDC80, RAD51, RRM2, SELE (includes EG:20339), SMC4, SOX7, TGM1, TK1, TOP2A, TYMS	ACTR1A, AR, AURKA, BCL2, BID, BIRC5, BUB3 (includes EG:12237), CBX5, CCNA2, CCNE1, CCNE2, CCNO, CDK1, CFLAR, CHEK1, CRYAB, CTSB, CYP27B1, DBF4 (includes EG:10926), DHFR, DNMT1, E2F2, ECT2, ENO2, FBXO5, FEN1, FGF2, FHL1 (includes EG:14199), FOXM1, GINS1, H19, HELLS, HN1, JMY, KIAA0101, LCK, MAD2L1, MCL1, MCM10 (includes EG:307126), MCM2, MCM5, MMP1 (includes EG:300339), MMP16, MYC, NDC80, PAWR, PPT2 (includes EG:100126133), RAD51, RBBP4, RPS16, RRM2, SELE (includes EG:20339), SMC4, SOX7, STAM, TGM1, TK1, TOP2A, TXNL1, TYMS, VCP, WWOX
**NfkB1-RelA**	complex			1.678	0.00126		**Act**	2.36	**1.06E-02**	CCL2, CCL5, CCND1, CXCL1, DEFB4A/DEFB4B, IL8, MMP1 (includes EG:300339), SELE (includes EG:20339)	**CCL2, CCL5, CXCL1, DEFB4A/DEFB4B, IL8, MMP1 (includes EG:300339), MYC, SELE (includes EG:20339)**
**STAT1**	TR	11.79		1.649	7E-12	**11.790**	**Act**	2.60	**1.65E-05**	APOBEC3G, APOE, BIRC5, CASP1, CCL19, CCL2, CCND1, CD274, CXCL10, DEFB4A/DEFB4B, FOS, GBP1, IDO1, IFI27, IFI35, IFI6, IL17A, IL1B, IL8, IRF1 (includes EG:16362), IRF7, IRF8, IRF9, ISG15, MMP9, MUC4, PTN, SERPINA3, SERPINB3, SERPINB4, SOAT1, SOCS1, TNFSF10, TRIM22, WARS	**BIRC5, CASP1, CCL2, CD274, CXCL10, DEFB103A/DEFB103B, DEFB4A/DEFB4B, GBP1, IFI27, IFI35, IFI6, IL8, IRF1 (includes EG:16362), IRF7, IRF8, IRF9, ISG15, MMP9, MYC, PMAIP1, PTN, SERPINA3, SERPINB3, SERPINB4, SOAT1, SOCS1, TLR3, TRIM22, TYMP, WARS**
**STAT3**	TR	7.47		1.829	1.7E-22	**7.470**	**Act**	2.96	**3.48E-15**	ANGPTL4, ARG2 (includes EG:11847), BCL2, BIRC5, CCL2, CCL5, CCNB1, CCND1, CCR5, CD274, CD83, CDK1, CEACAM1 (includes others), CEBPD, CTSB, CXCL10, CXCL13, CXCL2, CXCL9, FERMT2, FOS, HERC5, HK2, HMOX1, IFI27, IFI35, IFI44, IFI6, IFIH1, IFIT1, IFIT3, IL17A, IL4R, IL8, IRF1 (includes EG:16362), IRF7, ISG15, ISG20, LILRB2, MCL1, MMP9, MUC1, MX1, MX2, MYD88, NAMPT, OAS1, OAS2, OAS3, OASL, PLAU, PLAUR, PLSCR1, PTN, RAB27A, RORA, RORC, SERPINA3, SERPINB1, SERPINB3, SERPINB4, SERPINB9, SOCS3, SOD2, SP110, STAT1, STAT3, THBD, TNFSF10, TRIM14, TRIM22, WNT5A, XAF1	**ANGPTL4, ARG2 (includes EG:11847), BCL2, BIRC5, CCL2, CCL5, CCNB1, CD274, CD83, CDK1, CEACAM1 (includes others), CEBPD, CFLAR, CTSB, CXCL10, CXCL13, CXCL2, CXCL9, DNMT1, HERC5, HK2, HMOX1, IFI27, IFI35, IFI44, IFI6, IFIH1, IFIT1, IFIT3, IFIT5, IL4R, IL8, IRF1 (includes EG:16362), IRF7, ISG15, ISG20, LILRB2, MCL1, MMP9, MUC1, MX1, MX2, MYC, MYD88, NAMPT, OAS1, OAS2, OAS3, OASL, PLAU, PLAUR, PLSCR1, PMAIP1, PTN, RAB27A, RORA, SERPINA3, SERPINB1, SERPINB3, SERPINB4, SERPINB9, SOCS3, SOD2, SP110, STAT1, STAT3, THBD, THBS1, TRIM14, TRIM22, WNT5A, XAF1**
**NANOG**	TR					**-2.590**	**Act**	2.45	**2.42E-01**		**BIRC5, COL3A1, DLX5, GATA6, ISL1, LGALS1, LHX2, MAP1B, PDCD4, ZFHX3**
**NR0B2**	TR						**Act**	2.27	**4.78E-01**		**ESRRG, FOXO1 (includes EG:2308), PDK4, PPARGC1A**
