# Supplementary material for: Meta-Analysis Derived (MAD) Transcriptome of Psoriasis Defines the “Core” Pathogenesis of Disease
Source: PLoS One. 2012 Sep 5;7(9):e44274. doi: 10.1371/journal.pone.0044274 (PMC3434204; doi:10.1371/journal.pone.0044274)
Supplement: Table S6 — The 20 “classification” genes selected by MTGDR procedure. A. Estimated coefficients for each gene in each study. B. Misclassification error rates in each study and an overall error rate using a 5-fold cross-validation. (PDF) [file pone.0044274.s007.pdf]

A.

Symbol		S-F	Reischl	Gudjonsson	S-F+	Yao
ACTC1	actin, alpha, cardiac muscle 1	-0.1673	-0.1278	-0.3167	-0.6487	-0.1295
AKR1B10	aldo-keto reductase family 1, member B10 (aldose reductase)	0.0371	0.0459	0.0311	-0.0161	0.1221
CCL27	chemokine (C-C motif) ligand 27	-0.4934	-0.4076	-0.6941	-0.5561	-0.5388
CHRNA9	cholinergic receptor, nicotinic, alpha 9	0.3508	0.1513	0.2825	0.1221	0.5233
CLDN8	claudin 8	-0.1281	-0.0641	-0.084	-0.0546	-0.1192
CXCL10	chemokine (C-X-C motif) ligand 10	0.0265	0.0072	0.0712	0.0973	0.0478
CXCL9	chemokine (C-X-C motif) ligand 9	0.0291	0.019	0.0527	0.0574	0.0518
CYP2E1	cytochrome P450, family 2, subfamily E, polypeptide 1	0.044	0.0281	0.0947	0.2629	0.0402
IFI44L	interferon-induced protein 44-like	0.0334	0.0767	0.1869	0.2798	0.1733
KLRB1	killer cell lectin-like receptor subfamily B, member 1	0.0061	0.0023	0.006	0.0474	0.0182
KYNU	kynureninase	0.0535	0.0561	0.0277	0.0598	0.1029
MUC7	mucin 7, secreted	-0.0603	-0.0161	0.0024	-0.0273	-0.0287
NLRP2	NLR family, pyrin domain containing 2	0.0013	0.0015	-0.0029	0.0278	0.0023
POSTN	periostin, osteoblast specific factor	-0.099	-0.0702	-0.0847	-0.1676	-0.2085
RSAD2	radical S-adenosyl methionine domain containing 2	0.0811	0.0643	0.1298	0.248	0.0268
S100A12	S100 calcium binding protein A12	0.458	0.4196	0.6377	0.6108	0.6139
SERPINB3	serpin peptidase inhibitor, clade B (ovalbumin), member 3	0.0016	0.0017	6.00×10^{-4}	-0.0019	0.008
SERPINB4	serpin peptidase inhibitor, clade B (ovalbumin), member 4	0.0086	0.012	0.0435	0.0544	0.0215
TCN1	transcobalamin I (vitamin B12 binding protein, R binder family)	0.0401	0.062	0.0681	0.054	0.0558
VNN1	vanin 1	0.0419	0.0127	0.0199	0.1208	0.0547

B.

	Training			Cross-Validation		
	LS	NL	Error	LS	NL	Error
General	1	1	0.58%	2	3	1.3 %
S-F	0	0	0 %	0	0	0 %
T	0	0	0 %	0	0	0 %
S-F+	1	1	0.61 %	2	1	1.9 %
G	0	0	0 %	0	1	0.9 %
